# Unraveling the 17β-Estradiol Degradation Pathway in *Novosphingobium tardaugens* NBRC 16725

**DOI:** 10.3389/fmicb.2020.588300

**Published:** 2020-12-07

**Authors:** Juan Ibero, Beatriz Galán, Virginia Rivero-Buceta, José L. García

**Affiliations:** Department of Microbial and Plant Biotechnology, Centro de Investigaciones Biológicas Margarita Salas, Consejo Superior de Investigaciones Científicas, Madrid, Spain

**Keywords:** estrogens, catabolism, bacteria, biodegradation, 17β-estradiol, cytochrome P450, E1-hydroxylase, estrogen transport

## Abstract

We have analyzed the catabolism of estrogens in *Novosphingobium tardaugens* NBRC 16725, which is able to use endocrine disruptors such as 17β-estradiol, estrone, and estriol as sole carbon and energy sources. A transcriptomic analysis enabled the identification of a cluster of catabolic genes (*edc* cluster) organized in two divergent operons that are involved in estrogen degradation. We have developed genetic tools for this estrogen-degrading bacterium, allowing us to delete by site-directed mutagenesis some of the genes of the *edc* cluster and complement them by using expression plasmids to better characterize their precise role in the estrogen catabolism. Based on these results, a catabolic pathway is proposed. The first enzyme of the pathway (17β-hydroxysteroid dehydrogenase) used to transform 17β-estradiol into estrone is encoded out of the cluster. A CYP450 encoded by the *edcA* gene performs the second metabolic step, i.e., the 4-hydroxylation of estrone in this strain. The *edcB* gene encodes a 4-hydroxyestrone-4,5-dioxygenase that opens ring A after 4-hydroxylation. The initial steps of the catabolism of estrogens and cholate proceed through different pathways. However, the degradation of estrogens converges with the degradation of testosterone in the final steps of the lower catabolic pathway used to degrade the common intermediate 3aα-H-4α(3′-propanoate)7a-β-methylhexahydro-1,5-indanedione (HIP). The TonB-dependent receptor protein EdcT appears to be involved in estrogen uptake, being the first time that this kind of proteins has been involved in steroid transport.

## Introduction

Estrogens are C18 steroid hormones synthetized in animals through the elimination of cholesterol side chain and play several physiological roles (Ryan, 1982). The environmental release of estrogens is becoming an increasing public concern because they are important endocrine-disrupting compounds (EDCs) that persistently contaminate surface water, affecting the physiology of aquatic fauna and humans at extremely low concentrations (Teles et al., 2004). Therefore, the occurrence and abundance of estrogens in aquatic environments present a serious risk to public health (Barbosa et al., 2016). In addition, they have been classified by the World Health Organization as group 1 carcinogens (https://monographs.iarc.fr/list-of-classifications-volumes/). Among these compounds, the highest estrogenic activity is found in natural hormones like estrone (E1), 17β-estradiol (E2), and estriol (E3) and the synthetic estrogen 17α-ethinylestradiol (EE2).

The complete mineralization of estrogens to CO_2_ can be accomplished by a limited number of bacteria, mainly from the phyla Proteobacteria and Actinobacteria (Fujii et al., 2002; Fahrbach et al., 2006; Yu et al., 2007, 2016; Kurisu et al., 2010; Chen et al., 2017; Wang et al., 2019; Li et al., 2020). Although some biotransformation steps have been described in estrogen-degrading strains, the complete degradation pathway still remains unknown (Yu et al., 2013; Wang et al., 2014, 2018, 2019; Chen et al., 2017).

The degradation of A and B rings of steroids is achieved through different peripheral pathways depending on the compound, including the 9,10-*seco* pathway (for aerobic degradation of sterols and androgens), the 2,3-*seco* pathway (for the anaerobic degradation of sterols and androgens), and the 4,5-*seco* pathway (for aerobic degradation of estrogens) (Wang et al., 2014; Van Hamme et al., 2016; Chen et al., 2017). The anaerobic degradation of estrogens has been described in the *Denitratisoma* sp. strain DHT3 very recently (Wang et al., 2020). The ability to degrade estrogens anaerobically relies in the retroconversion of estrogens into androgens *via* a cobalamin-mediated methylation reaction (Wang et al., 2020).

**Figure 1:**
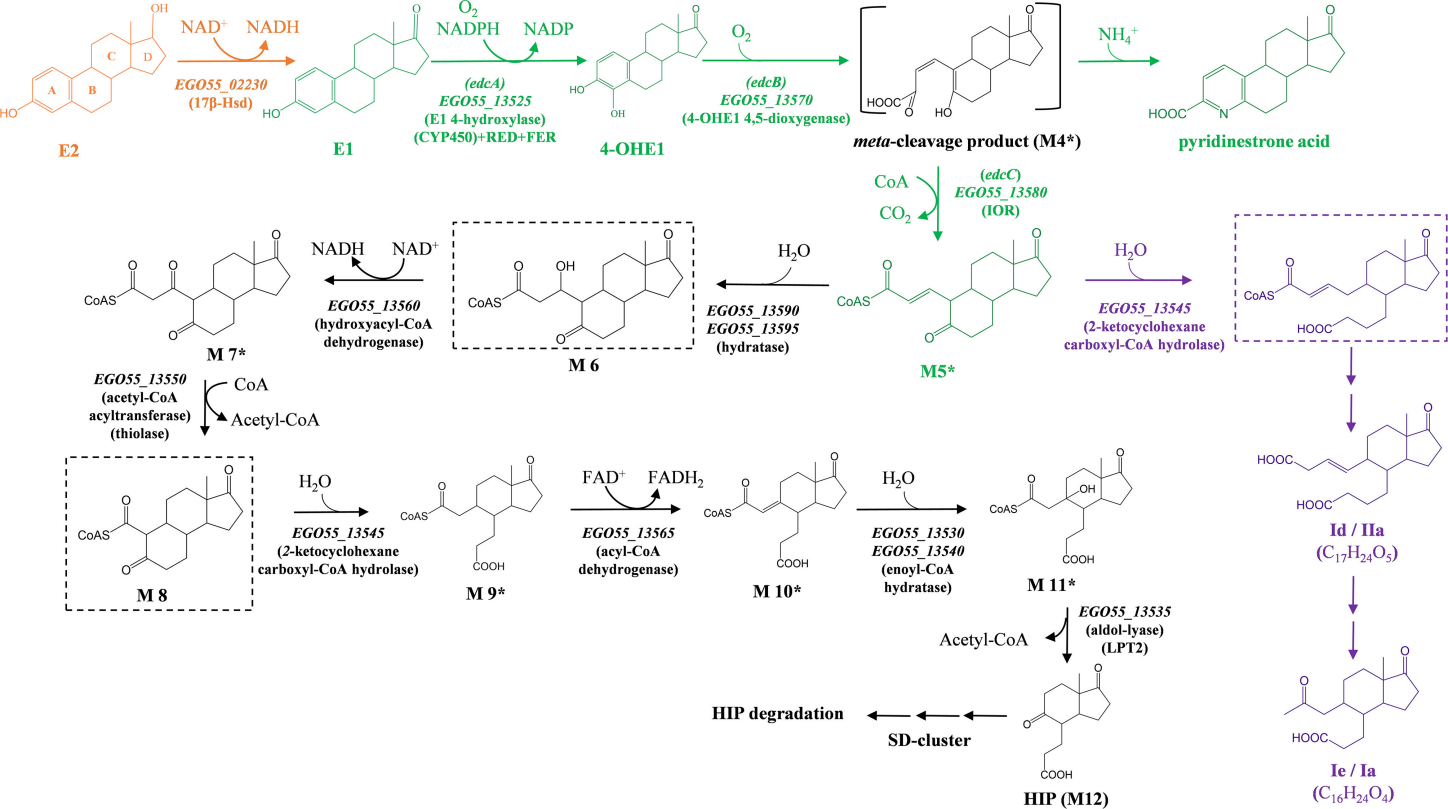
Proposed estrogen degradation pathway in *N. tardaugens* NBRC 16725. Compound names are indicated with an abbreviation: (E2) estradiol; (E1) estrone; (4-OHE1) 4-hydroxyestrone; (M5) 4-norestrogen-5(10)-en-3-oyl-CoA; (Ie/IIa) 3aα-*H*-4α-[3′-propanoic acid]-5β-[4′-but-3-enoic acid]-7aβ-methyl-l-indanone; (Id/Ia) 3aα-*H*-4α-[3′-propanoic acid]-5β-[2'-ketopropyl]-7aβ-methyl-l-indanone; and (HIP) 3aα-H-4α(3′-propanoate)-7a-β-methylhexahydro-1,5-indanedione. Enzyme names are (17β-Hsd) 3β, 17β-hydroxysteroid dehydrogenase; (EdcA) E1 4-hydroxylase; (EdcB) 4-OHE1 4,5-dioxygenase; and (EdcC) *meta*-cleavage product decarboxylase. The catalytic genes of *N. tardaugens* are indicated in italics, with the nomenclature EGO55_xxxxx. We indicate in orange the metabolic steps confirmed in our previous study (Ibero et al., 2019a); in green, those genes, enzymes, metabolites, and reactions covered in the present study; in purple, the proposed lateral pathway leading to dead-end products Ie/IIa and Id/Ia; and in black, metabolic steps described in previous studies (Chen et al., 2017; Wu et al., 2018). Only metabolites identified through mass spectrum are marked with asterisk, while the rest have been confirmed using NMR or authentic standards. Hypothetical metabolites are boxed in dashed-line squares.

For the aerobic degradation of estrogens, it has been proposed that the E2 degradation pathway (i.e., the 4,5-*seco* pathway) begins with 17β-dehydrogenation to render E1, followed by the oxygenolytic degradation of the aromatic ring A through the previous 4-hydroxylation and the subsequent *meta*-cleavage reaction (Chen et al., 2017; Figure 1). The *meta*-cleavage product of 4-hydroxyestrone is unstable and undergoes a recyclization with ammonia *via* a non-enzymatic reaction to produce the end product pyridinestrone acid (Chen et al., 2017) and was also detected as a metabolite derived from estrogen degradation in different bacteria (Coombe et al., 1966; Chen et al., 2018; Li et al., 2020). Based on transcriptomic analysis, two gene clusters (clusters I and II) have been identified in *Sphingomonas* sp. strain KC8 and proposed to be involved in the degradation of rings A and B of E1. A third cluster (III) is proposed for the degradation of C and D rings. Cluster I contains *oecA, oecB*, and *oecC* genes, which have been proposed to carry out the initial steps of the degradation pathway. However, only the functions of *oecA* and *oecC* gene products, coding a 17β-hydroxysteroid dehydrogenase (17βHSD) and 4-hydroxyestrone dioxygenase, respectively, have been confirmed by heterologous expression in *Escherichia coli* (Chen et al., 2017). Cluster II includes several putative β-oxidation-related genes, suggesting that CoA ester metabolites are likely to be involved in estrogen degradation. Further metabolite profile analyses enabled the prediction of some possible steps responsible for the aerobic biodegradation of natural estrogens (Wu et al., 2018). For instance, the detection of 3aα-H-4α(3′-propanoate)7a-β-methylhexahydro-1,5-indanedione (HIP) in *Sphingomonas* sp. KC8 growing on E2 pointed out the central role played by this metabolite, in which all the peripheral steroid degradation pathways known so far converge in a common central pathway (Horinouchi et al., 2006; Casabon et al., 2013; Barrientos et al., 2015; Wu et al., 2018).

One of the major drawbacks encountered in studies on the degradative pathways of estrogens in bacteria was the absence of genetic tools to manipulate those pathways by using genetic approaches. Thus, the availability of such genetic tools is a critical factor to further advance in the characterization of these pathways and their utilization for biotechnological purposes.

In this study, we used *Novosphingobium tardaugens* NBRC 16725 (formerly described as strain ARI-1), which was isolated in a sewage treatment plant in Tokyo (Japan) and is able to use sex hormones such as testosterone (TES), E2, E1, and E3 as sole carbon and energy sources (Fujii et al., 2002, 2003; Ibero et al., 2019a). Recently, we have reported the complete sequence and assembly of this E2-degrading bacterium genome into a single contig (Ibero et al., 2019b). In this study, we have performed transcriptomic analysis that enabled the identification of the catabolic genes involved in estrogen degradation. The development of genetic tools in this strain has allowed, for the first time, to delete some genes of the estrogen degradative pathway and confirm their precise role in estrogen catabolism. Furthermore, we have identified for the first time a CYP450 that performs the 4-hydroxylation of E1 in bacteria and a putative estrogen transport system.

## Results

### Whole Transcriptomic Analysis of *N. tardaugens* Grown on E2

To determine the expression of genes involved in the degradation of estrogens, we performed RNA-seq analyses in *N. tardaugens* cultured using PYR (control condition) or E2 as carbon sources. Differential expression analysis yielded 1,368 differentially expressed genes (DEGs) (from 3,980 total genes in genome), where 600 were upregulated and 768 were downregulated in E2 cultures compared to PYR [being fold change (FC) +2 or −2, respectively, the cutoff value] (Table S1), showing a noticeable contrast in differential expression pattern (Figure S1). The highest level of upregulation (FC > 10) was observed in 61 genes (Table S1), but other 88 genes were notably upregulated (5 < FC < 10) (Table S1). There are some similarities with the previously described transcriptomic data for this strain when grown on TES compared to PYR (Ibero et al., 2019a): a slight differential induction of genes between *EGO55_13695* and *EGO55_13795* (*SD* cluster), induction of the methylmalonyl degradation pathway gene cluster, and high expression levels of genes involved in cobalamin synthesis pathway (Table S1). In our previous work (Ibero et al., 2019a), we demonstrated that a 17βHSD, encoded by *EGO55_02230*, transformed E2 to E1, and we reported similar expression levels in TES to those now observed in E2-grown cells (Table S1), suggesting that its product could be responsible for the 17-dehydrogenation of both TES and E2. However, the differential characteristic of the E2 transcriptome is the upregulation of a region in the genome (≈19 kb) comprising genes *EGO55_13520* to *EGO55_13600* that will be named estrogen degradation cluster (*edc*) henceforth.

### Annotation of the *edc* Cluster

**Figure 2:**
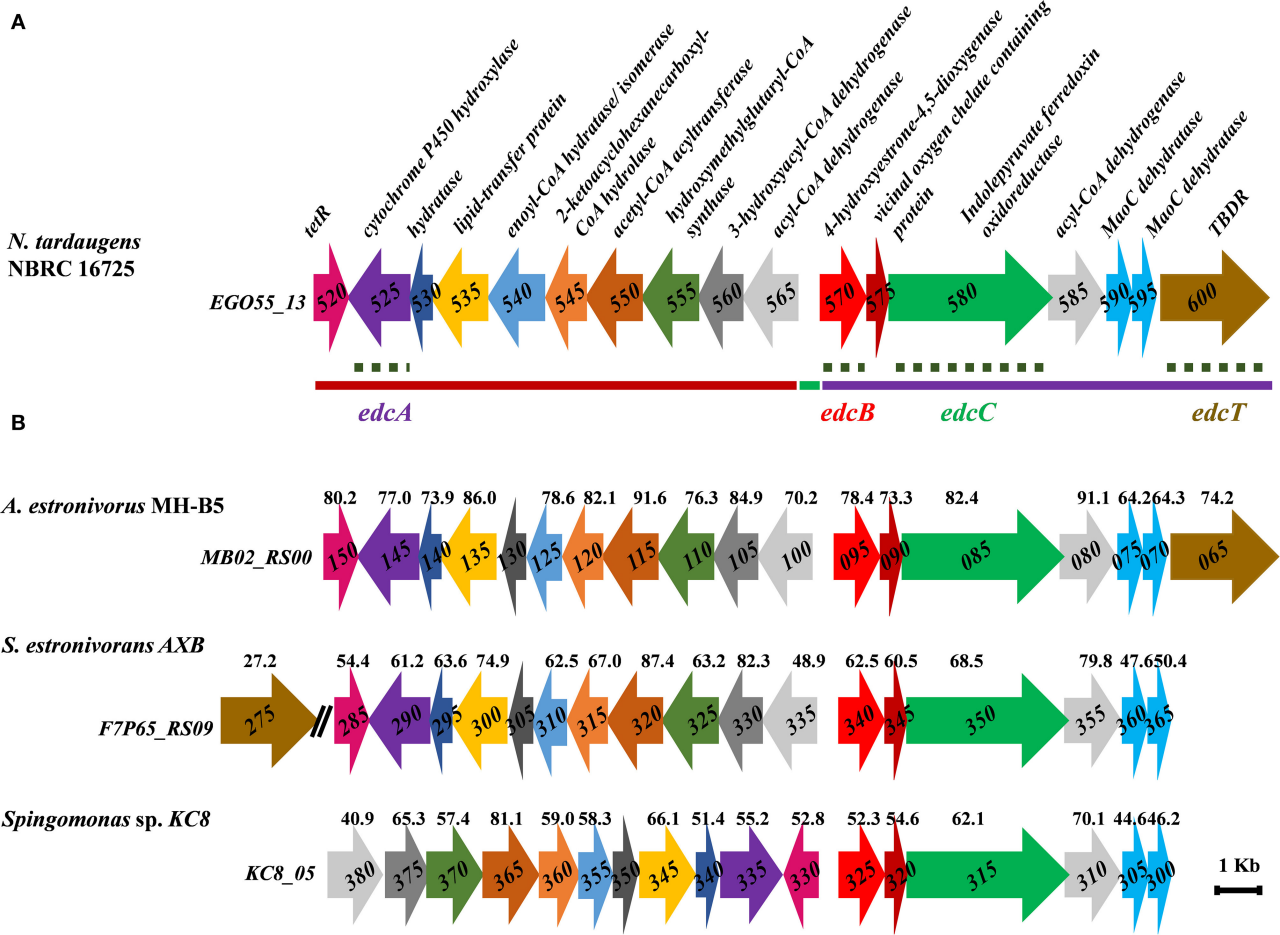
**(A)** Scheme of the *N. tardaugens* NBRC 16725 estrogen degradation cluster (*edc*) (accession number CP034179). Regions deleted by double homologous recombination are depicted with a line: ΔProm (green), ΔOpA (red), and ΔOpB (purple). Gene deletions by double homologous recombination that allowed function confirmation in this study are marked with a dashed line (*edcA, edcB, edcC*, and *edcT*). **(B)** Estrogen degradation gene cluster of *A. estronivorus* MH-B5 (accession number NZ_JRQQ01000001.1), *S. estronivorans* AXB (accession number NZ_LFCT01000011.1), and *Sphingomonas* sp. KC8 gene cluster II (accession number CP016306). Genes encoding the same function are pictured in the same color and % identity of protein products toward those from *N. tardaugens* is shown.

The E2 degradation cluster of *N. tardaugens* NBRC 16725 occurs within a 52.8-kb stretch of the chromosome *EGO55_13520–EGO55_13795* (Figure 2). This region is composed of two large gene clusters: (i) the *edc* cluster consists of 17 genes which have been shown to be upregulated in E2 in this work (Table S1) and (ii) the *SD* cluster, which consists of 26 genes that are slightly upregulated in E2 and TES (Table S1) (Ibero et al., 2019a). The 16 genes located within the SD and *edc* gene clusters encode a wide variety of proteins, including one regulator, one outer membrane transporter, and several other enzymes related to metabolism (Table S1) that will require a further study to demonstrate their possible involvement in steroid metabolism.

The *edc* cluster from *N. tardaugens* is similar to cluster II of *Sphingomonas* sp. KC8 (Chen et al., 2017). Moreover, other clusters similar to the *edc* cluster can be found in other sequenced bacteria such as *Altererythrobacter estronivorus* MH-B5 (Qin et al., 2016) and *Sphingobium estronivorans* AXB (Qin et al., 2020; Figure 2). It is interesting to notice that, in spite of the similarities, the orientations of the genes as well as the gene composition of the operons of the clusters are not identical, which might render some clues about the functionality of the genes.

The *edc* cluster of *N. tardaugens* consists of two divergent putative operons, OpA (*EGO55_13525*–*EGO55_13565*) and OpB (*EGO55_13570*–*EGO55_13600*), and another divergently expressed gene *EGO55_13520*, which encodes a putative TetR transcriptional regulator of the pathway that is currently under study. Operons OpA and OpB are divergently transcribed and separated by an intergenic region of 137 bp that putatively encloses both promoter sequences (Figure 2).

The OpA operon encodes a putative cytochrome P450 hydroxylase (CYP450) (*EGO55_13525*; *edcA*) whose function in E2 degradation was unknown. There is also a group of genes that encode a hydratase (*EGO55_13530*; *chsH2-like*), a lipid-transfer protein (*EGO55_13535*), an enoyl-CoA hydratase/isomerase (*EGO55_13540*), a 2-ketocyclohexanecarboxyl-CoA hydrolase (BadI-like, thiolase) (*EGO55_13545*), an acetyl-CoA acyltransferase (thiolase) (*EGO55_13550*), a 3-hydroxy-3-metylglutaryl-CoA synthase (HMG-CoA synthase) (*EGO55_13555*), a 3-hydroxyacyl-CoA dehydrogenase (*EGO55_13560*), and an acyl-CoA dehydrogenase (*EGO55_13565*). Some of these enzymes are homologous to those involved in lipid metabolism.

It is interesting to notice that the *EGO55_13540* gene of *N. tardaugens* is larger than the homologous genes shown in Figure 2. It appears to be a perfect in-frame fusion of the homologous genes *KC8_05355* and *KC8_05350* from *Sphingomonas* sp. KC8 (Figure 2). This fusion is not a sequence artifact since it was confirmed by several methods including genome sequencing by Illumina and PacBio as well as by the transcriptome reads of this paper. Thus, the *EGO55_13540* gene might have a double function.

The OpB operon encodes a putative 4-hydroxyestrone-4,5-dioxygenase (*EGO55_13570*; *edcB*) that shares 52.3% identity with the well-characterized 4-hydroxyestrone-4,5-dioxygenase (OecC) from *Sphingomonas* sp. KC8 (Chen et al., 2017). This enzyme belongs to the glyoxalase-like family and has been proposed to perform the *meta*-cleavage reaction of 4-OHE1. The *EGO55_13575* gene encodes a protein that contains a vicinal oxygen chelate (VOC) domain and appears to be a member of a large family of enzymes that catalyze a highly diverse set of reactions, containing enzymes such as glyoxalases I, extradiol dioxygenases, bleomycin resistance proteins, fosfomycin resistance proteins, and methylmalonyl-CoA epimerases. The *EGO55_13580* (*edcC*) gene encodes a member of the indolepyruvate ferredoxin oxidoreductase (IOR) family. EdcC shares a 62.1% sequence identity with the protein product of the *KC8_05315* gene, belonging to the family of IORs. This enzyme has been proposed for the decarboxylation of the *meta*-cleavage product in *Sphingomonas* sp. KC8, based on the activity of other enzymes of this family (Wu et al., 2018). In the metabolism of aromatic amino acids, IORs are responsible for the oxidative decarboxylation of 2-oxo acids, generating the corresponding acetyl-CoA derivative (Mai and Adams, 1994; Kletzin and Adams, 1996; Schut et al., 2001). The C3 and C4 carbons of the *meta*-cleavage product have a 2-oxo acid structure, and this compound can be the substrate for an enzyme of this family, such as EdcC. The *EGO55_13585* gene encodes an acyl-CoA dehydrogenase. *EGO55_13590* and *EGO55_13595* genes encode two MaoC hydratases. The *EGO55_13600* gene encodes a putative TonB-dependent receptor (TBDR).

The previously described SD cluster was postulated to be involved in TES degradation in *N. tardaugens*, containing genes responsible for the steroid CD-ring degradation, i.e., HIP degradation (Ibero et al., 2019a), that has been proposed as the convergent metabolite of steroid degradative pathways (Horinouchi et al., 2006; Casabon et al., 2013; Barrientos et al., 2015; Wu et al., 2018).

### Functional Analysis of the *edc* Cluster

The possibility of transforming *N. tardaugens* has allowed us to use genetic tools to analyze the functionality of the genes involved in estrogen degradation. So far, none of the bacteria able to degrade estrogens has been genetically manipulated, and thus, these tools have open a new scenario to precisely dissect the pathway. To prove the true involvement of the *edc* gene cluster, comprised of OpA and OpB operons, in E2 catabolism, we have constructed two large knockout mutants. Firstly, we have deleted the entire intergenic region, which includes both putative operon promoters (*P*_*a*_*-P*_*b*_) yielding the ΔProm strain: The ΔProm mutant will be in principle unable to express all the *ecd* genes. Secondly, OpA and OpB operons were deleted, yielding ΔOpA and ΔOpB mutants, respectively (Figure 2).

**Figure 3:**
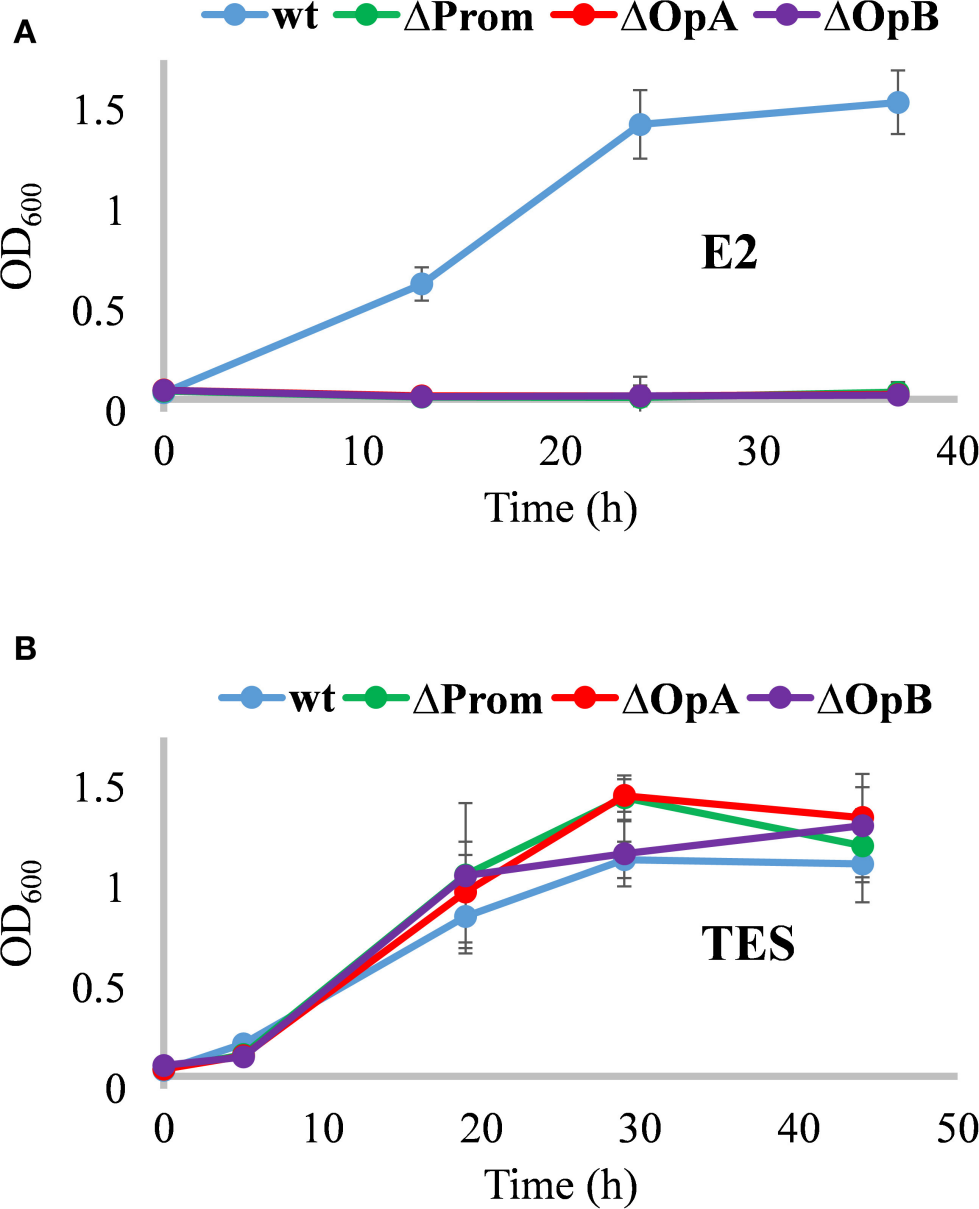
Growth phenotype of *N. tardaugens* NBRC 16725 (blue), ΔProm (green), ΔOpA (red), and ΔOpB (purple) strains. Growth curves (OD 600 nm) in minimal medium M63 supplemented with **(A)** 2 mM E2 and **(B)** 1.89 mM TES (data corresponding to biological triplicates and error bars show standard deviation).

As expected, ΔProm, ΔOpA, and ΔOpB mutants were unable to grow on estrogens (Figure 3A), but they were able to grow on NB rich medium, and more importantly, in minimal media containing TES as sole carbon and energy sources (Figure 3B). This result confirmed, for the first time with a genetic approach, that the *edc* cluster is critical for the degradation of estrogens. The OpA and OpB operons have no redundancy, and thus, they cannot be substituted by other operons in the genome. In addition, it also confirmed that none of the *edc* genes are required for the degradation of androgens like TES in *N. tardaugens*.

The ΔProm, ΔOpA, and ΔOpB mutants were further grown in rich medium supplemented with E2 to study the possible accumulation of steroidal intermediates. Interestingly, the ΔProm mutant accumulated E1 (Figure S2), suggesting that 17βHSD activity is encoded outside the *edc* cluster. In fact, we have not identified a 17βHSD encoding gene in the *edc* cluster, suggesting that this activity was encoded in other chromosomal locus. This result is in agreement with the observation that a 17βHSD encoding gene is found out of cluster II of *Sphingomonas* sp. KC8 (Chen et al., 2017). This result also demonstrates that, without the enzymes encoded in the *edc* cluster, the metabolism of E1 cannot progress any further, suggesting that the second proposed step in the estrogen degradation pathway, i.e., the hydroxylation of E1, is more probably encoded within the cluster. The accumulation of E1 was also detected in the ΔOpA (Figure S2), suggesting that the enzyme responsible for the hydroxylation of E1 should be encoded in the OpA operon. The organic fraction extracted from cultures of ΔProm and ΔOpA mutants did not show any other intermediate different from E1, suggesting that E1 cannot be modified by other enzymes of *N. tardaugens* apart from those encoded by the *edc* cluster.

**Figure 4:**
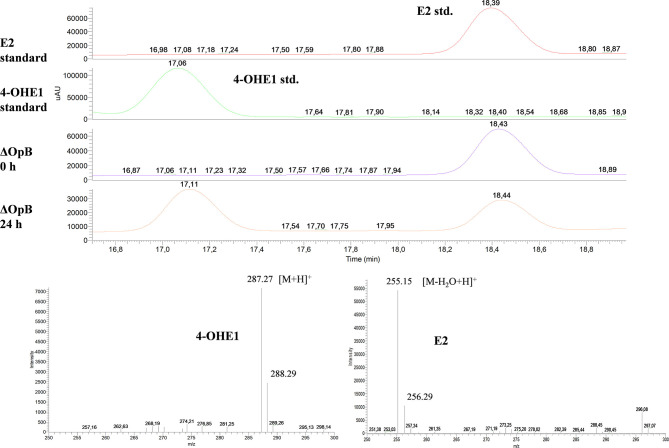
HPLC-MS analysis of the organic phase extracted from cultures of *N. tardaugens* ΔOpB growing on NB rich medium supplemented with 2 mM E2. DAD (*diode array detector*) chromatograms at 50–600 nm of the E2 and 4-OHE1 standards and ΔOpB samples at 0 and 24 h and mass spectra of the peaks detected in ΔOpB at 24 h.

Interestingly, HPLC-MS analysis of cultures of ΔOpB mutant identified a compound with an *m*/*z* of 286, sharing elution time and *m*/*z* with 4-OHE1 commercial standard (Figure 4). The OpB operon contains the *EGO55_13570* gene, annotated as a putative 2,3-dihydroxybiphenyl 1,2-dioxygenase, which shows a 52.3% identity to *oecC* (4-hydroxyestrone-4,5-dioxygenase), which transforms 4-OHE1 into the *meta*-cleavage product (Chen et al., 2017). Therefore, the accumulation of 4-OHE1 might be explained by the absence of this dioxygenase activity. Besides, the detection of 4-OHE1 in ΔOpB mutant cultures suggests that the hydroxylase activity of E1 is encoded in the OpA operon.

### Analysis of *N. tardaugens* Mutants Carrying Specific Deletions of Some *edc* Genes

To determine the function of some *edc* genes in E2 metabolism, three genes were deleted. Using this genetic approach, we have checked the ability of the mutants to grow on E2, compared to the wild-type strain, and investigated the accumulation of intermediates that might render information about the functionality of these genes. In this sense, ΔedcA, ΔedcB, and ΔedcC mutant strains were constructed, in which cytochrome P450 (*EGO55_13525; edcA*), putative extradiol dioxygenase (*EGO55_13570; edcB*), and putative indolepyruvate ferredoxin oxidoreductase (*EGO55_13580; edcC*), which might be responsible for the three first steps of the E1 degradation pathway, were deleted, respectively (Figure 2).

**Figure 5:**
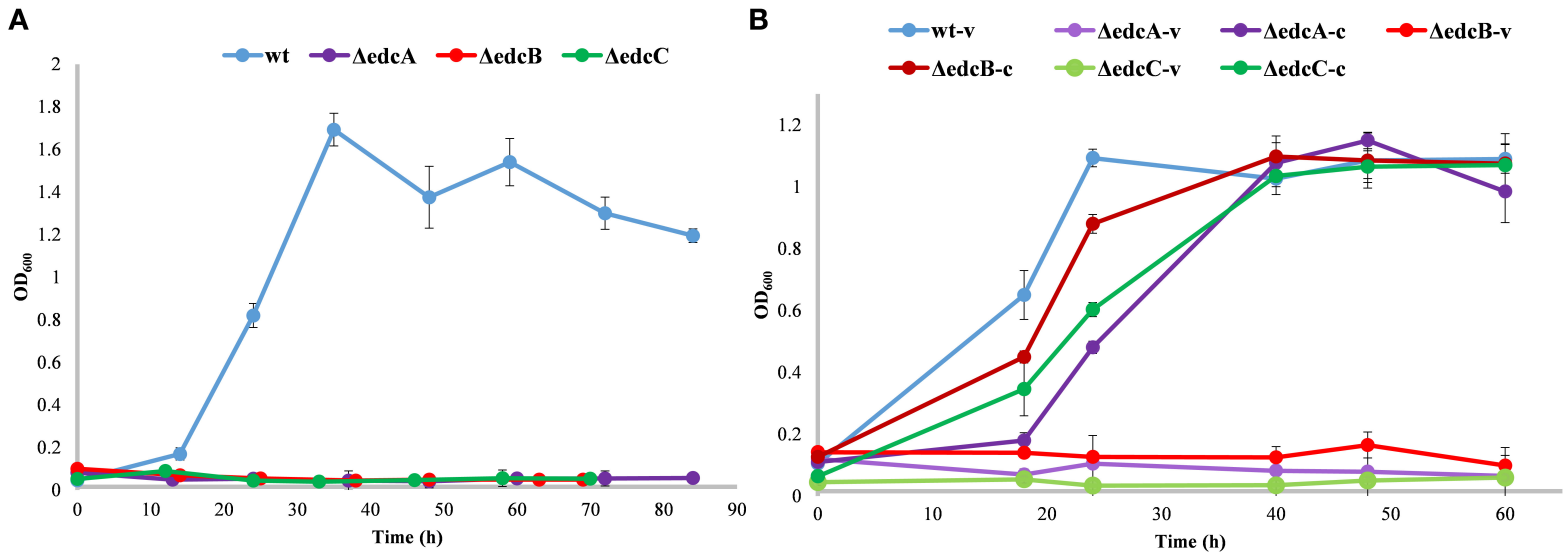
Growth curves (OD_600_) in minimal medium M63 supplemented with 2 mM E2. **(A)** Growth of *N. tardaugens* NBRC 16725 (blue), ΔedcA (purple), ΔedcB (red), and ΔedcC (green). **(B)** Growth of *N. tardaugens* NBRC 16725 and the mutant strains with pSEVA23PlexA, (-v), and complemented with the corresponding deleted gene, (-c). Data corresponding to biological triplicates and error bars show standard deviation.

ΔedcA, ΔedcB, and ΔedcC mutants showed an impaired growth on E2 as sole carbon and energy source (Figure 5A). Significantly, the ability of the mutants to grow on E2 was restored by the trans complementation of the mutants expressing the deleted genes on a plasmid (Figure 5B), demonstrating that the deletions have only affected the specific genes.

When these mutants were cultured in NB rich medium supplemented with E2 to detect the accumulation of intermediates in the culture, the HPLC-MS analysis showed a characteristic metabolite accumulation pattern for each mutant.

**Figure 6:**
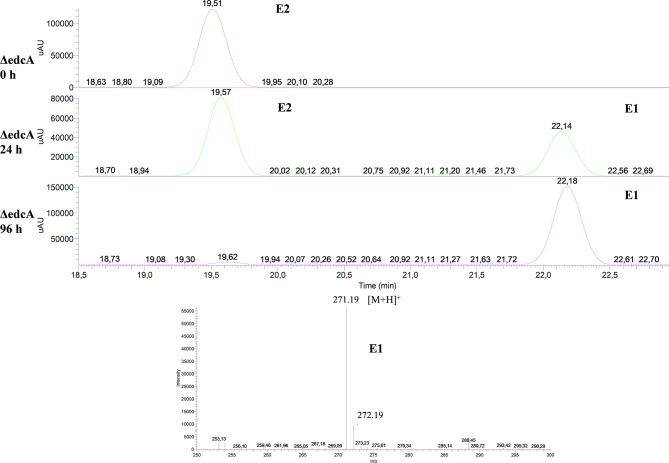
HPLC-MS analysis of the organic phase extracted from cultures of *N. tardaugens* ΔedcA in NB rich medium supplemented with 2 mM E2. UV chromatograms at 282 nm of the samples at 0, 24, and 96 h and mass spectrum of the peak detected at 22.18 min (E1).

**Figure 7:**
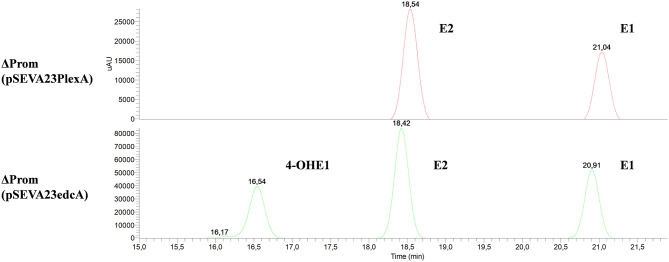
HPLC-MS analysis of the organic phase extracted from cultures from *N. tardaugens* ΔProm (pSEVA23PlexA) and *N. tardaugens* ΔProm (pSEVA23edcA) growing on NB rich medium supplemented with 2 mM E2. UV chromatograms at 282 nm of the samples at 47 h.

Firstly, the ΔedcA mutant accumulated E1 (Figure 6), suggesting that the *EGO55_13525* gene could be involved in E1 hydroxylation. To confirm this hypothesis, the ΔProm mutant strain, lacking the ability to express all the genes from the *edc* cluster, was complemented with a plasmid expressing the *edcA* gene. Interestingly, when the ΔProm (pSEVA23ecdA) strain grew in NB rich medium supplemented with E2, we detected the production of 4-OHE1 (Figure 7). This result supports the hypothesis that cytochrome P450 (CYP450) EdcA is responsible for the hydroxylation of E1. This assumption was not unexpected if we consider that E1 and E2 hydroxylation is carried out in mammals by a CYP450 (Lønning et al., 2011). However, our result does not agree with the proposal of Chen et al. (2017) who have suggested that the *oecB* gene, encoding a putative monooxygenase, is responsible for E1 hydroxylation in *Sphingomonas* KC8.

**Figure 8:**
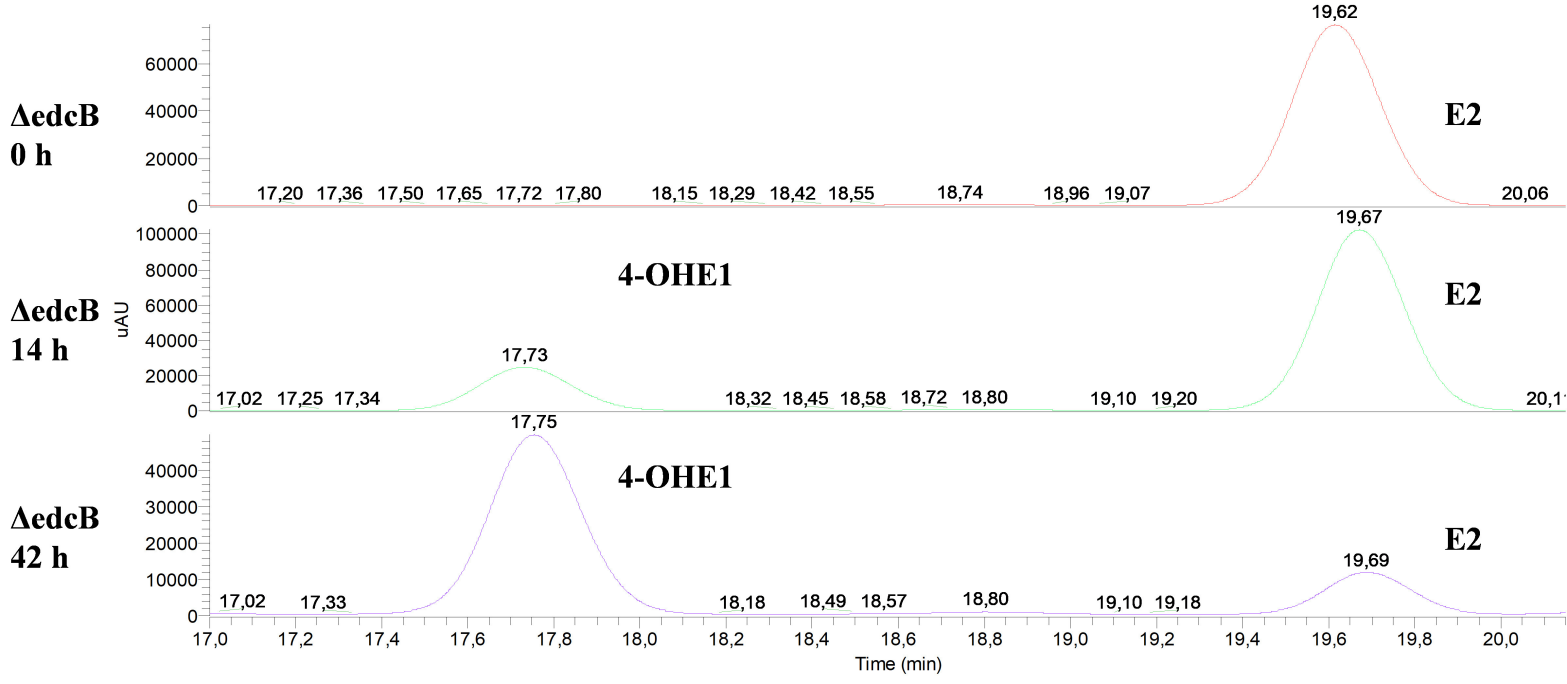
HPLC-MS analysis of the organic phase extracted from cultures of *N. tardaugens* ΔedcB in NB rich medium supplemented with 2 mM E2. UV chromatograms at 282 nm of the samples at 0, 14, and 42 h.

Secondly, the ΔedcB mutant strain accumulated 4-OHE1 (Figure 8), supporting the hypothesis that the *EGO55_13570* gene encodes a 4-hydroxyestrone-4,5-dioxygenase, in this case in agreement with the proposal of Chen et al. (2017).

**Figure 9:**
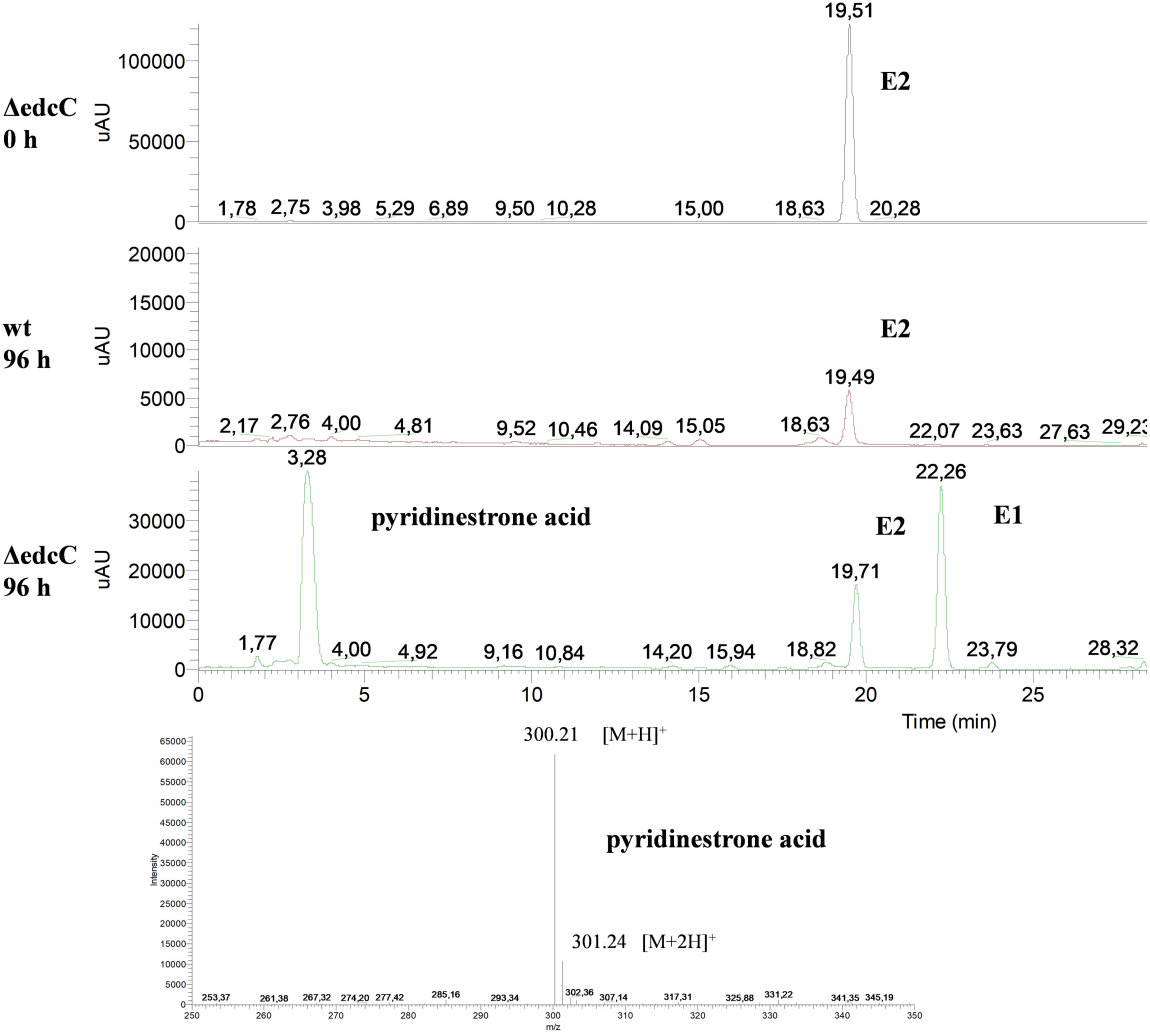
HPLC-MS analysis of the organic phase extracted from cultures of *N. tardaugens* ΔedcC in NB rich medium supplemented with 2 mM E2. UV chromatograms at 282 nm of the samples at 0 and 96 h for ΔedcC strain and 96 h for wt strain and mass spectrum of the peak detected at 3.28 min (pyridinestrone acid).

Finally, the ΔedcC mutant accumulated a metabolite of *m*/*z* 300 that could correspond to pyridinestrone acid (Figure 9). This compound was described as a side product in *Sphingomonas* sp. KC8 that spontaneously derives from the *meta*-cleavage product in the presence of ammonia (Chen et al., 2017). This result suggests that the *meta*-cleavage product of ring A should be the substrate of EdcC. In agreement with this result, the homologous indolepyruvate ferredoxin oxidoreductase present in cluster II of KC8 has been proposed as an enzyme that decarboxylates the *meta*-cleavage product of ring A and, at the same time, ligates a CoA molecule to the resulting new carboxylic residue to facilitate further degradation (Wu et al., 2018). In the absence of this enzyme, the resulting *meta*-cleavage product of ring A cannot progress, and therefore, it is spontaneously cyclized with ammonium to render a pyridine molecule.

### EdcA Catalyzes E1 Hydroxylation *in vitro*

**Figure 10:**
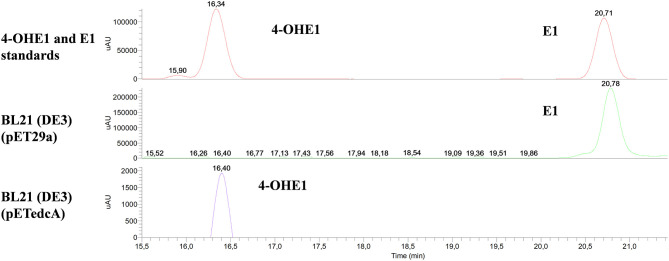
HPLC-MS analysis of the organic phase extracted from the enzymatic assay using BL21 (DE3) (pETedcA) cell crude extract. UV chromatograms at 282 nm of 4-OHE1 and E1 standards and the product from the enzymatic assay using BL21 (DE3) (pET29a) and BL21 (DE3) (pETedcA) cell crude extract.

To determine the activity of CYP450 EdcA *in vitro*, the *edcA* gene was cloned into *E. coli* pET29a expression vector generating pETedcA plasmid that allows the overexpression of EdcA in *E. coli* BL21 (DE3) as a C-terminal His-tagged fusion protein. The overproduction of the tagged protein in the presence of isopropyl-β-D-thiogalactopyranoside (IPTG) was observed by SDS-PAGE in the protein extracts (Figure S3). The enzymatic activity of CYP450 was examined *in vitro*, and the oxidation of E1 after 30 min of incubation using *E. coli* BL21 (DE3) (pETedcA) crude extracts was observed, as judged from the appearance of a new peak in the HPLC chromatograms (Figure 10). The relative retention times (Rt) and mass spectrum of the product were consistent with those of 4-OHE1 (Rt 16.10 min, *m*/*z* 287). When the hydroxylase activity of EdcA was tested on other putative steroidal substrates like E2 and E3, the enzyme was able to hydroxylate E2, but not E3 (Figure S4).

### Analyzing the Role of the *edcB* Gene by Additional Genetic Studies

To prove that the *edcB* gene encodes a 4-hydroxyestrone-4,5-dioxygenase, we have performed additional genetic studies. In this sense, *EGO55_13570* (*edcB*) and *EGO55_13525* (*edcA*) genes were cloned together in a pSEVA23PlexA vector under the control of the *P*_*b*_ promoter, which includes the intergenic region of the *edc* cluster, generating the pSEVA237-Pb-edcAB plasmid (Figure S5) that was transformed into ΔProm mutant, yielding the ΔProm (pSEVA237-Pb-edcAB) strain. To be used as controls, we have also constructed the strain ΔProm (pSEVA237-Pb-edcA), carrying only the *ecdA* gene under the control of the *P*_*b*_ promoter and the strain ΔProm (pSEVA237PlexA) carrying the empty plasmid without any gene. It should be noticed that the strain ΔProm (pSEVA237-Pb-edcA) is very similar to the strain ΔProm (pSEVA23ecdA) mentioned above, but in the last case, the *edcA* gene is under control of the *P*_*lexA*_ promoter.

**Figure 11:**
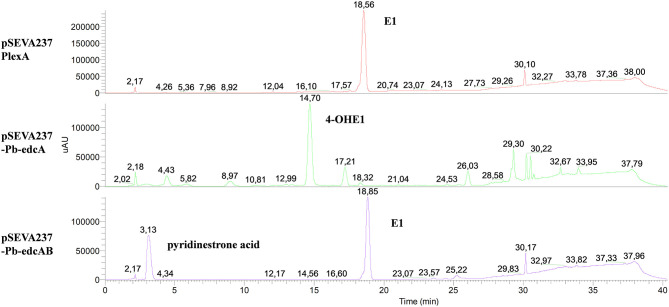
HPLC-MS analysis of the organic fraction extracted from cultures of *N. tardaugens* ΔProm (pSEVA23-PlexA), ΔProm (pSEVA237-Pb-edcA), and ΔProm (pSEVA237-Pb-edcAB) growing on NB rich medium supplemented with 2 mM E1 from samples at 38 h. DAD (*diode array detector*) chromatograms at 50–600 nm.

ΔProm (pSEVA237-Pb-edcAB), ΔProm (pSEVA237-Pb-edcA), and ΔProm (pSEVA237PlexA) strains were grown on NB rich medium supplemented with E1, and the organic phase was extracted to analyze the accumulation of metabolites in the cultures. The HPLC-MS analysis of ΔProm (pSEVA237-Pb-edcAB) cultures showed a peak at 3.13 min, which did not appear in the ΔProm (pSEVA23PlexA) and ΔProm (pSEVA237-Pb-edcA) strains used as controls (Figure 11). As expected, the ΔProm (pSEVA237-Pb-edcA) accumulates 4-OHE1, confirming the results shown by the ΔProm (pSEVA23edcA) strain (Figure 7). The peak at 3.13 min accumulated in the ΔProm (pSEVA237-Pb-edcAB) strain corresponds to a compound of *m*/*z* 300, consistent with pyridinestrone acid (Chen et al., 2017). This result strongly supports that EdcB is using 4-OHE1 as substrate and it is required for opening ring A.

### Analyzing the Role of the *edcC* Gene by Additional Genetic Studies

**Figure 12:**
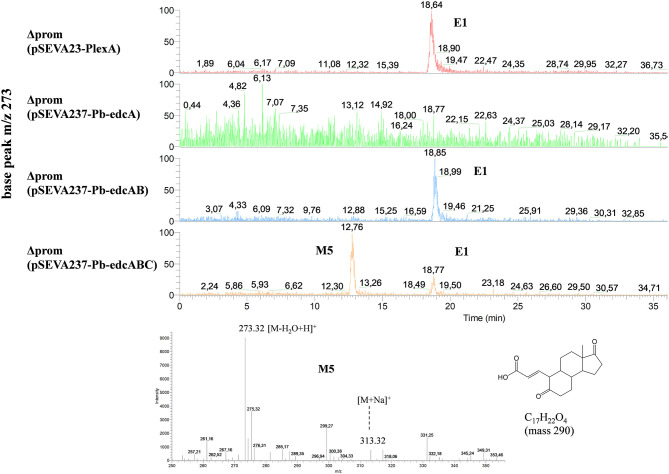
HPLC-MS analysis of the organic phase extracted from cultures of *N. tardaugens* ΔProm (pSEVA237-Pb-edcABC) in NB rich medium supplemented with 2 mM E1 at 38 h. Total ion chromatogram (TIC) of base peak *m*/*z* 273 of ΔProm (pSEVA23-PlexA), ΔProm (pSEVA237-Pb-edcA), ΔProm (pSEVA237-Pb-edcAB), and ΔProm (pSEVA237-Pb-edcABC) and mass spectrum of the peak observed at 12.76 min (4-norestrogen-5(10)-en-3-oic acid).

To demonstrate that the *edcC* gene is required in the next step of estrogen degradation, after cleavage of 4-OHE1, we have cloned the *edcC* gene into plasmid pSEVA237-Pb-edcAB delivering the pSEVA237-Pb-edcABC plasmid (Figure S5), which was transformed into the ΔProm strain. The resulting strain, ΔProm (pSEVA237-Pb-edcABC), was grown on NB rich medium supplemented with E1, and the extracted organic fraction was analyzed by HPLC-MS. The rationale of this experiment is based on the fact that the accumulation of pyridinestrone acid resulting from the consecutive activities of EdcA and EdcB should be reduced or even avoided in the presence of the following decarboxylating activity of EdcC. Figure 12 shows the accumulation of a compound with an elution time of 12.76 min that is only detected in the organic fraction from the culture of the ΔProm (pSEVA237-Pb-edcABC) strain (Figure 12). This peak might correspond to an ion (M−H_2_O+H)^+^ of a compound of mass 290 without one water molecule. This mass is in agreement with the mass of the deconjugated (i.e., without CoA) M5 compound (Figure 1), proposed as an intermediate derived from the decarboxylation and activation with CoA of the *meta*-cleavage product in *Shingomonas* sp. KC8 (Wu et al., 2018). The accumulation of this compound in the culture of the ΔProm (pSEVA237-Pb-edcABC) strain supports the role of EdcC in the decarboxylation of the *meta*-cleavage product derived from the dioxygenase activity of EdcB in *N. tardaugens*.

### Analyzing the Role of *edcA* in E3 Degradation

**Figure 13:**
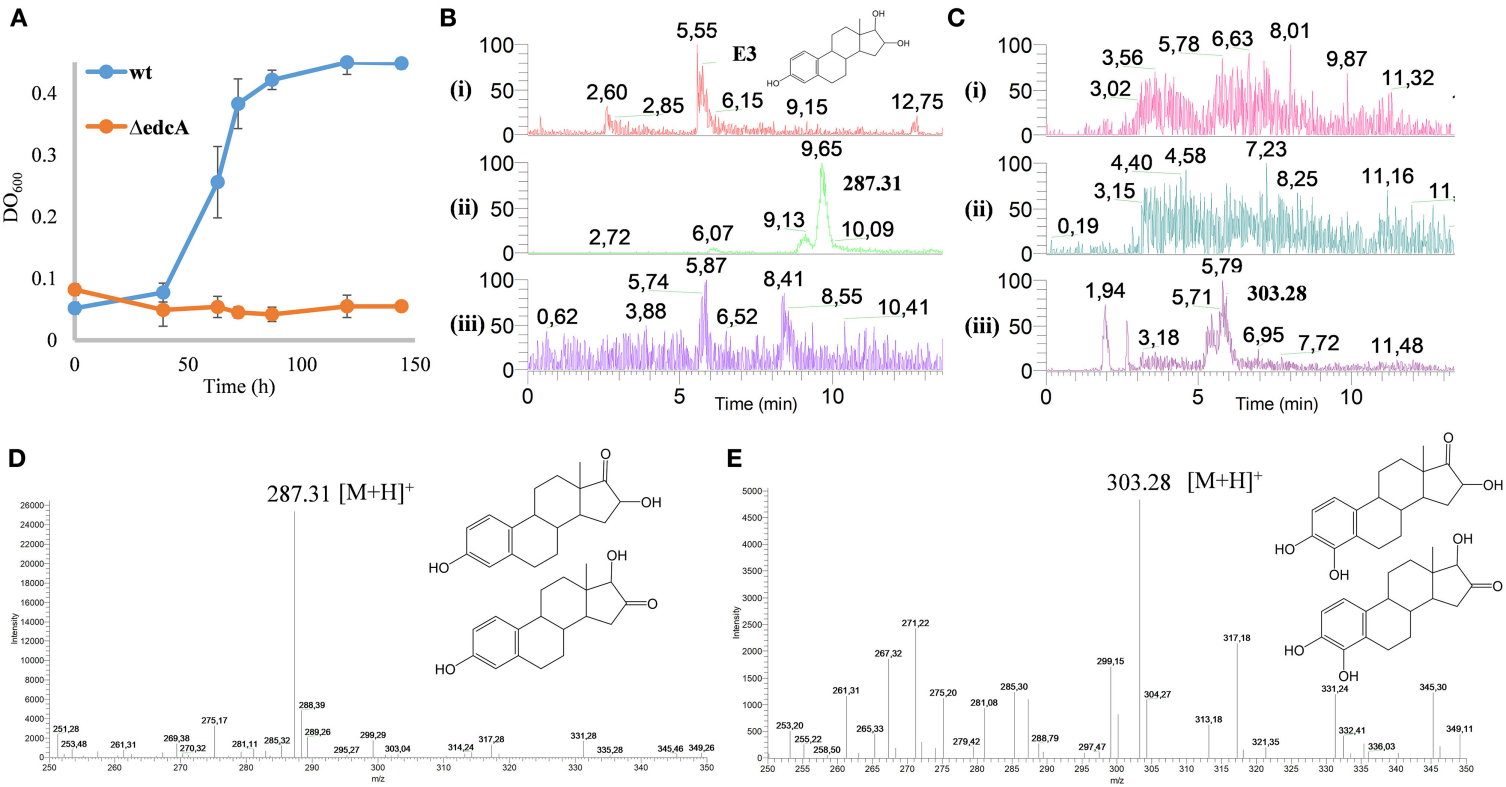
**(A)** Growth curves (OD_600_) on M63 minimal medium with 1.89 mM E3 of *N. tardaugens* NBRC 16725 and ΔedcA. Data corresponding to biological triplicates and error bars show standard deviation. HPLC-MS analysis of the organic fraction extracted from cultures of *N. tardaugens* ΔProm (pSEVA23-PlexA) and *N. tardaugens* ΔProm (pSEVA23-edcA) in NB rich medium supplemented with 1.89 mM E3, at 38 h. Total ion chromatogram (TIC) of base peak *m*/*z* **(B)** 287 and **(C)** 303. The samples correspond to the control of non-inoculated medium, (i); *N. tardaugens* ΔProm (pSEVA23-PlexA) strain, (ii); and *N. tardaugens* ΔProm (pSEVA23-edcA) strain, (iii). Mass spectrum of the peak observed at **(D)** 9.65 min and **(E)** at 5.79 min.

*N. tardaugens* is able to metabolize E3 using it as a sole carbon and energy source to grow, but the mechanisms involved in this catabolism remain unknown (Fujii et al., 2002). To investigate E3 catabolism, the wild-type and ΔedcA mutant strains of *N. tardaugens* were cultivated in E3 as a sole carbon and energy source. The growth curve showed that the ΔedcA mutant was unable to grow on E3 (Figure 13A), suggesting that *edcA* is essential in the degradation of E3 in *N. tardaugens*. However, this experiment did not allow us to know if EdcA could hydroxylate E3 or if E3 has to be previously transformed into other intermediate to be hydroxylated and degraded afterwards. In fact, we have not observed *in vitro* the hydroxylation of E3 by CYP450 (see above).

To investigate these options *in vivo*, ΔProm (pSEVA23PlexA) and ΔProm (pSEVA23ecdA) strains were grown on NB rich medium supplemented with E3, and the organic fraction extracted from the culture medium was analyzed by HPLC-MS.

The accumulation of a compound eluting at 9.65 min of *m*/*z* 287 (M+H)^+^ was observed in the organic fraction from the culture of the ΔProm (pSEVA23PlexA) strain. However, this compound was not observed in the ΔProm (pSEVA23-ecdA) strain, expressing *edcA* (Figure 13B). The detected ion could correspond to a compound with a keto group of mass 286, resulting from the dehydrogenation of one hydroxyl group of E3 at position 16 (16-keto-E2) or 17 (16α-OH-E1) (Figure 13D). We believe that most probably this compound should correspond to 16α-OH-E1 by the action of a 17βHSD. This reaction should be similar to the reaction that transforms E2 into E1. Nevertheless, we cannot ascertain that the process is performed by the same enzyme, since there are several putative 17βHSD encoded in the genome of *N. tardaugens*.

On the other hand, the ΔProm (pSEVA23ecdA) strain accumulated a compound with *m*/*z* of 303 (M+H)^+^ at 5.79 min (Figure 13C). This compound has a difference in mass of 16 that could correspond most probably to the addition of a hydroxyl group at C4 in 16α-OH-E1. Unfortunately, since we do not have a standard of this compound, we could not test the hydroxylation of 16α-OH-E1 by CYP450 *in vitro*.

All these results suggest that E3 and E2 are poor or null substrates for CYP450 *in vitro*; however, it can hydroxylate E1 (*in vivo* and *in vitro*) and 16α-OHE1 (*in vivo*) reinforcing the idea that the keto group at C17 is required by CYP450 EcdA to carry out the C4 hydroxylation of estrogens. Moreover, these results suggest that the *edc* cluster is involved in the degradation of E3.

### Analysis of E2 Transport

**Figure 14:**
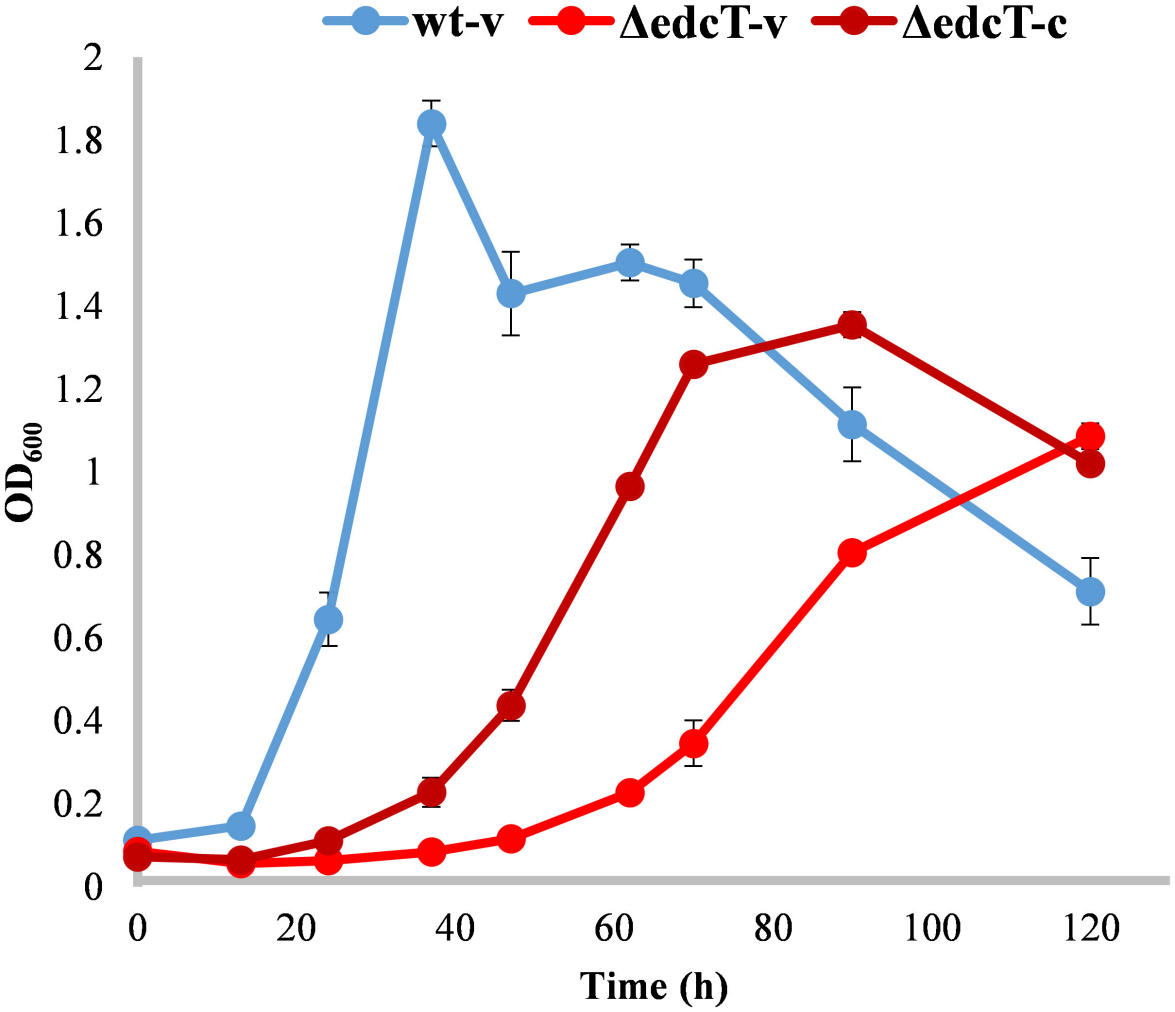
Growth curves (OD_600_) of *N. tardaugens* on M63 minimal medium supplemented with 2 mM E2 of NBRC 16725 and ΔedcT harboring the pSEVA23PlexA plasmid, (-v), and complemented with the deleted gene, (-c). Data corresponds to biological triplicates and error bars show standard deviation.

The *edcT* (*EGO55_13600*) gene, located at the end of the OpB operon (Figure 2), encodes a putative TonB-dependent receptor, whose expression is upregulated 91-fold in the presence of E2. TBDRs are bacterial outer membrane proteins that bind and transport ferric chelates, called siderophores, as well as vitamin B_12_, nickel complexes, and carbohydrates (Noinaj et al., 2010). To study the function of the putative TonB-dependent receptor EdcT on E2 catabolism, the *EGO55_13600* gene was deleted (Figure 2). The resulting ΔedcT mutant showed a decrease in growth rate and an increased lag phase with respect to the wild-type strain (Figure 14), suggesting that the TonB-dependent receptor protein could be involved in the transport of E2 into the cell. This result is also supported by the partial reversion of this behavior achieved by the ΔedcT (pSEVA23edcT) strain expressing the *edcT* gene cloned in plasmid pSEVA23 (Figure 14). This is, to the best of our knowledge, the first experimental result that links the transport of steroids to a TonB-dependent receptor.

## Discussion

In this work, we have used different experimental approaches to investigate the aerobic catabolism of estrogens in *N. tardaugens*. The global transcriptomic analysis in E2-grown cells has allowed us to identify the *edc* cluster, responsible for the aerobic degradation of A and B rings of E2 in this bacterium. The expression of the *edc* genes showed a remarkable induction upon growth on E2 (FC ranging from 16.6 to 91.7) that corresponded to the highest level of expression observed in the whole transcriptome. Besides the *edc* cluster, the induction of the methylmalonyl degradation pathway gene cluster supports the formation of propionyl-CoA in the lower HIP degradation pathway, due to the lack of methylcitrate cycle genes in the *N. tardaugens* genome. Moreover, the upregulation of genes involved in the cobalamin synthesis pathway is consistent with the requirement of this cofactor by the methylmalonyl-CoA mutase, which is upregulated in the presence of E2.

The data presented in this work have been analyzed in the light of the results obtained in *Sphingomonas* strain KC8 (Chen et al., 2017; Wu et al., 2018). Here, we propose an E2 catabolic pathway in *N. tardaugens* (Figure 1) including the degradation of E2 to HIP, since this compound is a common intermediate in steroid catabolism in all bacteria studied so far (Horinouchi et al., 2006; Casabon et al., 2013; Barrientos et al., 2015; Wu et al., 2018). The gene annotation of the *edc* cluster has allowed us to ascribe the genes to the proposed steps of the pathway with the exception of *EGO55_13*555 and *EGO55_13*575 (Figure 1). *EGO55_13*555 encoded a protein homologous to HMG-CoA synthases. These enzymes catalyze the condensation of acetyl-CoA with acetoacetyl-CoA. An anabolic reaction like this one does not appear to make sense in the estrogen degradative pathway. A phylogenetic analysis of this gene revealed that, although it can be classified as HMG-CoA synthase, it belongs to a different group in the current tree of HMG-CoA synthases (Figure S6). This finding suggests that this enzyme does not have a condensation activity and might have other activities (e.g., thiolase). In this sense, these enzymes belong to a thiolase superfamily including biosynthetic (HMG-CoA synthases, chalcone synthases, polyketide synthases and related enzymes) or degradative thiolases (Heath and Rock, 2002). *EGO55_13*575 encodes a protein member of a large family of enzymes containing a VOC domain that catalyze a highly diverse set of chemistries, some of them oxidative reactions (He and Moran, 2011). Enzymes in this VOC superfamily are represented by four families: isomerase, nucleophilic addition, extradiol dioxygenase, and α-keto acid oxygenase (He and Moran, 2011). Among those, the protein encoded by *EGO55_13*575 does not seem to be involved in nucleophilic addition (fosfomicyn resistance) nor isomerase (glyoxylase or methylmalonyl-CoA epimerase) reactions. Although the methylmalonyl pathway is proposed to be involved in propionyl-CoA (derived from HIP degradation) metabolism, the position of *EGO55_13575* between genes involved in ring A opening and decarboxylation suggests that it might act in earlier steps of the pathway. Whereas, other functions performed by VOC proteins, i.e., extradiol dioxygenase or α-keto acid oxygenase, seem to fit better the oxidation processes expected in this upper pathway, the diol structure on aromatic ring A is cleaved by EdcB, so the latter seems to be the only reasonable option. In this sense, the enzyme could be involved in reducing the length in carbons of the alkyl chain generated after ring cleavage, allowing the conversion of Id to Ie, for instance (Figure 1).

The aerobic catabolism of E2 starts with the dehydrogenation of the hydroxyl group at position 17β to produce E1. The *N. tardaugens* genome includes 16 homologous proteins encoding putative 17β-HSDs, and none of them are encoded within the *edc* cluster (Ibero et al., 2019a). Probably, such diversity of enzymes makes it evolutionary unnecessary to include this enzyme in the steroid degradative clusters that require a C17 oxidation. In this work, we have demonstrated that the 17β-HSD responsible to transform E2 into E1 is encoded outside the *edc* cluster since E1 was accumulated in some of the mutant strains where the E2 catabolism was impaired. In this sense, the *oecA* gene from *Sphingomonas* sp. KC8, encoding a 17β-HSD activity, is also located outside cluster II (Chen et al., 2017). 17β-HSDs are very common enzymes among bacteria and catalyze the dehydrogenation of different types of 17β-hydroxysteroids in steroid metabolism. There are some 17-HSDs that transform E2 to E1, homologous to the 17-HSD encoded by *EGO55_02230* in *N. tardaugens*, i.e., OecA from *Sphingomonas* sp. KC8 encoded by *KC8_09390* (20.09%) and the enzymes from *Pseudomonas putida* SJTE-1, 3-oxoacyl-ACP reductase, encoded by *A210_09220* (34.06%) (Wang et al., 2018), and 17β-HSD, encoded by *A210_19955* (37.90%) (Wang et al., 2019).

The E2 degradation pathway continues with 4-hydroxylation of E1 (Coombe et al., 1966). In *Sphingomonas* sp. KC8, transcriptomic analyses revealed the upregulation of *oecB* located within cluster I that encodes a putative flavin-dependent monooxygenase (OecB), and therefore, it was proposed to catalyze the 4-hydroxylation of E1, although this biochemical reaction was not demonstrated (Chen et al., 2017). Although *EGO55_13440* of *N. tardaugens*, homologous to *oecB*, was also upregulated in the E2-grown cells (8.0-fold change), the metabolites accumulated by ΔProm, ΔOpA, and ΔOpB mutant strains revealed that E1 hydroxylation activity was encoded in the OpA region. Although we cannot exclude that *EGO55_13440* could contribute to E1 hydroxylation in some growing conditions, we looked for a gene coding for this activity within the *edc* cluster. In this sense, the OpA operon encodes a putative CYP450 hydroxylase (*EGO55_13525*; *edcA*) that is upregulated 67.7-fold on the presence of E2, which was a clear candidate to carry out this biochemical step. CYP450s are enzymes involved in the oxidative degradation of many compounds, and they are particularly well-known for their role in the degradation of environmental toxins and mutagens. In mammals, endogenous estrogens, such as E2 and E1, undergo extensive oxidative metabolism (namely, hydroxylation and keto formation) at various positions, catalyzed by many different CYP450 isoforms present mostly in the liver (Thomas and Potter, 2013). In this work, this hypothesis was confirmed by *in vitro* assays and using *N. tardaugens* mutants. To the best of our knowledge, the conversion of E1 to 4-OHE1 catalyzed by a bacterial CYP450 has not been described so far. Interestingly, the *ecd* cluster does not contain genes encoding the required ferredoxin (Fd) and ferredoxin reductase (FdR), suggesting that these proteins are encoded in other loci. There are several genes that can be annotated to encode Fds (*EGO55_00615, EGO55_03165, EGO55_03280, EGO55_04875, EGO55_05120, EGO55_08480, EGO55_08640, EGO55_13190*) and FdRs (*EGO55_01285, EGO55_05820, EGO55_06710, EGO55_09415*); however, none of them were overexpressed upon growth on E2 (Table S1). Nevertheless, the number of read counts proves that some of them (i.e., three Fds, *EGO55_08480, EGO55_08640* and *EGO55_13190*, and two FdRs, *EGO55_01285* and *EGO55_09415*) are constitutively expressed and could be part of the redox-partner system of EdcA.

Although EdcA can also hydroxylate E2 *in vitro*, it does not seem to be the preferred substrate since the activity was very low when compared with that on E1. On the other hand, EdcA was unable to hydroxylate E3 *in vitro*, since as mentioned above, the presence of the keto group at C17 might be a strong requirement for EdcA activity. In accordance with *in vitro* results, while none hydroxylated derivatives of E3 have been reported in estrogen-degrading bacteria so far, only one study has reported the detection of 4-OHE2, and it was in resting cell transformations of E2, in the presence of the *meta*-cleavage inhibitor 3-chlorocatechol, by the environmental isolate *Sphingomonas* sp. ED8 (Kurisu et al., 2010).

The ΔedcA mutant strain did not grow on E3 and accumulates a compound that presumably corresponds to 16α-OHE1 (Figure 13) suggesting that E3 should be previously oxidized to 16α-OHE1 by a 17β-HSD coding in the *N. tardaugens* genome. The transient detection of 16α-OHE1 during growth on minimal medium with E3 was previously reported in aerobic cultures of the aquifer sand isolate *Agromyces* sp. LHJ3 strain and identified with authentic standards by LC-MS (Ke et al., 2007).

EcdA activity seems to be a bottleneck of the pathway, in accordance with the widespread transient detection of E1 as intermediate metabolite during E2 degradation. In agreement with this proposal, while the complete transformation of E2 to E1 has been reported during treatment of waste water spiked with E2 in constructed wetlands and in activated sludge (Ternes et al., 1999; Lee and Liu, 2002; Hashimoto and Murakami, 2009; Dai et al., 2016; Kopperi et al., 2016; Kassotaki et al., 2019), it has been observed that the disappearance/biotransformation of E1 was one order of magnitude lower than that of E2 (Ogunlaja and Parker, 2015).

Subsequently, we have shown that A ring cleavage at C4–C5 is catalyzed by a 4-OHE1 4,5-dioxygenase (EdcB) and the resulting *meta*-cleavage product appears to be the substrate for the putative indolepyruvate ferredoxin oxidoreductase (EdcC) as it was described before for other estrogen-degrading microorganisms (Coombe et al., 1966; Chen et al., 2017; Wu et al., 2018; Li et al., 2020).

Thereafter, several reactions occur involving the activity of β-oxidation-like enzymes such as enoyl-CoA hydratases, hydroxyacyl-CoA dehydrogenases, acyl-CoA dehydrogenase, thiolases, and aldolase to form HIP (Figure 1). It is important to notice that the pathway proposed in this work for the degradation of E2 does not contain some of the intermediates detected by other authors, such as compounds Id and Ie (Chen et al., 2018), correlating with IIa and Ia (Coombe et al., 1966), respectively. This pathway was constructed assuming a minimal number of steps according to the genes present in the *edc* cluster. We cannot exclude that some of these intermediates might result from an alternative opening of ring B just after the formation of 4-norestrogen-5(10)-en-3-oyl-CoA and not at the end of the pathway. Thus, we can envision that the 2-ketocyclohexanecarboxyl-CoA hydrolase (*EGO55_13545*) could act also on this compound generating the compounds Id and Ie, also named IIa and Ia, detected by Chen et al. (2018) and Coombe et al. (1966), respectively. Depending on the specificity of the enzymes and the accumulation of the intermediates during estrogen degradation, it would be possible to find different intermediates that are released to the culture medium as dead-end acidic products. These products cannot be incorporated in the pathway since they lose the CoA and they will require not only to be uptaken but also to bind a new CoA molecule to enter the pathway. It is known that the accumulation of a large amount of CoA intermediates within the cell might cause toxic stress (Brass, 1994). Thus, the release of CoA by unspecific thiolases of some pathway intermediates can be used by the cells in order to reduce this stress and recover some CoA for other metabolic functions.

On the other hand, we would like to point out that β-oxidation-like enzymes have been proven to be responsible for cholate degradation in some Proteobacteria (Barrientos et al., 2015; Holert et al., 2016), and it could be envisioned that these enzymes could be also involved in cholate degradation by *N. tardaugens*, since this strain is able to grow on cholate as a sole carbon and energy source (data not shown). However, our results suggest that cholate and estrogen degradation in *N. tardaugens* is carried out by different pathways. The *edc* cluster of *N. tardaugens* was not induced in cholate, and ΔProm, ΔOpA, or ΔOpB mutant strains were not impaired to grow on cholate as a sole carbon and energy source (data not shown). Furthermore, in *Sphingomonas* sp. KC8, the genes of cluster II are only involved in estrogen degradation, as the strain is unable to use cholate as a sole carbon source (Chen et al., 2017).

The final phase of estrogen degradation that encompasses the HIP degradation pathway follows a series of biochemical reactions involving enzymes similar to those for β-oxidation of fatty acids that in *N. tardaugens* are encoded in the SD cluster involved in the degradation of TES (Ibero et al., 2019a). Three pieces of evidence support that *N. tardaugens* uses the same gene products to degrade the C and D rings of both androgens and estrogens: (i) the SD cluster is upregulated in the presence of both E2 and TES compared with pyruvate; (ii) many of the genes contained in the SD cluster do not have other homologs in the *N. tardaugens* genome; and (iii) the predicted CD-ring degradation genes are highly conserved among different genera of steroid-metabolizing bacteria (Horinouchi et al., 2012, 2019; Holert et al., 2016; Van Hamme et al., 2016; Crowe et al., 2017).

Finally, the transport of steroids has been poorly studied in bacteria, and it was mainly limited to the transport of cholesterol or cholate in Gram-positive bacteria (Mohn et al., 2008; Pandey and Sassetti, 2008; Klepp et al., 2012; Haußmann et al., 2013; García-Fernández et al., 2017). The transport of steroids in Gram-negative bacteria poses a great challenge due to the presence of the outer membrane where a lipopolysaccharide leaflet on the outer surface limits transport through passive diffusion (Plésiat and Nikaido, 1992). Thus, only one study covers this topic in the Gram-negative, anaerobic cholesterol-degrading bacteria, *Sterolibacterium denitrificans* DSMZ 13999 (Lin et al., 2015). Whereas in Gram-positive bacteria ABC (ATP-binding cassette) transporters are responsible for cholesterol (Pandey and Sassetti, 2008; García-Fernández et al., 2017) and cholate transport (Haußmann et al., 2013), in *S. denitrificans*, it is a FadL-like system that carries out cholesterol transport (Lin et al., 2015). Thus, our studies on the role of EdcT open a new scenario in this field. The ΔedcT strain generated in this work showed a significant decrease in growth rate and an increased lag phase with respect to the wild-type strain (Figure 14), suggesting that the TonB-dependent receptor protein could be involved in estrogen uptake *in N. tardaugens*. So far, TBDRs have been proved to be involved in the transport of siderophores, vitamin B_12_, nickel complexes, carbohydrates (Noinaj et al., 2010), and also of aromatic compounds derived from lignin (Fujita et al., 2019), but to our knowledge, this would be the first time that the TonB-dependent receptor protein is linked to steroid transport.

The results presented in this work and the tools developed to manipulate *N. tardaugens* will pave the way to use this organism as a model to further investigate the steps of estrogen metabolism that render HIP intermediate. The regulatory and transport systems identified in this microorganism will be further studied since they open the possibility for developing regulatory systems and biosensors responding to estrogens. Moreover, taking into account that this bacterium is naturally able to degrade estrogens, androgens, and other steroid molecules, it can be used as an industrial chassis to expand its catabolic properties to degrade other abundant xenobiotic endocrine disruptors. Therefore, our aim for future studies is to investigate the possibility of using *N. tardaugens* to produce biofilters for remediation of contaminated water.

## Materials and Methods

### Chemicals

Testosterone (TES), 17β-estradiol (E2), estrone (E1), 4-hydroxyestrone (4-OHE1), cholate, pyruvate (PYR), chloroform, ethanol, sulfuric acid, and acetonitrile were purchased from Merck KGaA Sigma (Darmstadt, Germany). Randomly methylated β-cyclodextrin (TRMB-T randomly methylated BCD) (CDX) was purchased from Cyclodex (Alachua, United States). Other chemicals and reagents were purchased from Merck KGaA Sigma (Darmstadt, Germany).

### Strains and Growth Media

Bacterial strains and plasmids used in this study are listed in Table 1. *N. tardaugens* NBRC 16725 (strain ARI-1) was purchased from the Leibniz-Institut DSMZ-type culture collection. This strain and its mutants were cultured at 30°C in an orbital shaker at 200 rpm. Nutrient broth (NB) (Difco) was used as rich medium to grow this strain. Minimal medium M63 [KH_2_PO_4_ (136 g/L), (NH_4_)_2_SO_4_ (20 g/L), FeSO_4_·7H_2_O (5 mg/L), pH 7.0] was supplemented with 0.39 mM CaCl_2_, 1 mM MgSO_4_, and the appropriate carbon source concentration. We used a carbon equimolar concentration for each substrate tested. Steroids and PYR stock solutions were prepared in PBS buffer and 70 mM CDX so the final carbon concentration in the culture was 36 mM in 13.33 mM CDX. *E. coli* DH10B, *E. coli* BL21 (DE3), and *E. coli* HB101 strains were grown at 37°C in an orbital shaker at 200 rpm in lysogeny broth (LB) medium (Sambrook and Russell, 2001). The appropriate antibiotics, i.e., chloramphenicol (34 μg/ml), kanamycin (50 μg/ml), or rifampicin (50 μg/ml), were added when needed.

**Table 1:** Bacterial strains and plasmids used in this study.

**Strains**	**Genotype and characteristics**	**Source/references**
***N. tardaugens***		
NBRC 16725	Wild-type strain (ARI-1)	Fujii et al., 2003
Rf^R^	Rf^r^ strain efficient for conjugation	Ibero et al., 2019a
ΔProm	*N. tardaugens* NBRC 16725 where the intergenic region between *EGO55_13565* and *EGO55_13570* has been deleted	This study
ΔOpA	*N. tardaugens* NBRC 16725 where the region *EGO55_13565-EGO55_13520* has been deleted	This study
ΔOpB	*N. tardaugens* NBRC 16725 where the region *EGO55_13570-EGO55_13600* has been deleted	This study
ΔedcA	*N. tardaugens* NBRC 16725 Δ*EGO55_13525*	This study
ΔedcB	*N. tardaugens* NBRC 16725 Δ*EGO55_13570*	This study
ΔedcC	*N. tardaugens* NBRC 16725 Δ*EGO55_13580*	This study
ΔedcT	*N. tardaugens* NBRC 16725 Δ*EGO55_13600*	This study
***E. coli***		
DH10B	F^−^, *mcr*A, Δ(*mrr hsdRMS*-*mcrBC*), Φ80d*lacZ*ΔM15, Δ*lacX74, deoR, recA1, araD139*, Δ(*ara-leu*)7697, *galU, galK*, λ^−^, *rpsL, endA1, nupG*	Invitrogen
BL21 (DE3)	F^−^, *ompT, hsdSB* (*rB*^−^*mB*^−^) *gal, dcm* λDE3 (harboring gene 1 of the RNA polymerase from phage T7 under the PlacUV5 promoter)	Sambrook and Russell, 2001
HB101	*supE44 ara14 galK2 leuB lacY1* Δ(*gpt*-*proA*)62 *rps*L20 *xyl-5 mtl-1 recA13* Δ(*mcrC*-*mrr*) *hsdS20* (rB^−^mB^−^) Sm^R^	Sambrook and Russell, 2001
**Plasmids**	**Description**	**References**
pRK600	Cm^r^ ColE1*oriV* RP4 *oriT*; helper plasmid in triparental mattings	Sharma and Signer, 1990
pK18*mob*sacB	Km^r^, ColE *oriV*, Mob+, *lacZα, sacB*; vector for allelic exchange homologous recombination mutagenesis	Schafer et al., 1994
pK18Prom	pK18*mob*sacB derivative containing fragments UP-Prom and DOWN-Prom used for ΔProm mutant construction, Km^R^	This study
pK18OpA	pK18*mob*sacB derivative containing fragments UP-OpA and DOWN-OpA used for ΔOpA mutant construction, Km^R^	This study
pK18OpB	pK18*mob*sacB derivative containing fragments UP-OpB and DOWN-OpB used for ΔOpB mutant construction, Km^R^	This study
pK18edcA	pK18*mob*sacB derivative containing fragments UP-edcA and DOWN-edcA used for *EGO55_13525* deletion, Km^R^	This study
pK18edcB	pK18*mob*sacB derivative containing fragments UP-edcB and DOWN-edcB used for *EGO55_13570* deletion, Km^R^	This study
pK18edcC	pK18*mob*sacB derivative containing fragments UP-edcC and DOWN-edcC used for *EGO55_13580* deletion, Km^R^	This study
pK18edcT	pK18*mob*sacB derivative containing fragments UP-edcT and DOWN-edcT used for *EGO55_13600* deletion, Km^R^	This study
pSEVA237PlexA	Km^R^, *oriV* (pBBR1), constitutive expression of *gfp* gene under the control of the *PlexA* promoter	Fernández et al., 2014
pSEVA23PlexA	pSEVA237PlexA where *gfp* was deleted using *Xba*I–*Spe*I restriction enzymes and served as empty vector	This study
pSEVA23edcA	pSEVA237PlexA where the *gfp* gene was replaced by *EGO55_13525*	This study
pSEVA23edcB	pSEVA237PlexA where the *gfp* gene was replaced by *EGO55_13570*	This study
pSEVA23edcC	pSEVA237PlexA where the *gfp* gene was replaced by *EGO55_13580*	This study
pSEVA23edcT	pSEVA237PlexA where the *gfp* gene was replaced by *EGO55_13600*	This study
pSEVA237PbPlexA	pSEVA237PlexA where the promoter region *P_*b*_* was cloned	This study
pSEVA237Pb-edcA	pSEVA237Pb containing the *EGO55_13520* gene	This study
pSEVA237Pb-edcAB	pSEVA237Pb containing the *EGO55_13520* and *EGO55_13570* genes	This study
pSEVA237Pb-edcABC	pSEVA237Pb containing the *EGO55_13525, EGO55_13570*, and *EGO55_13580* genes	This study
pET-29a(+)	Cloning and expression vector, Km^r^, *ori*ColE1, *P_*T*7_* promoter	Novagen
pET29edcA	pET-29 containing the *EGO55_13525* gene	This study

### Estrogen Biotransformation Process

*N. tardaugens* NBRC 16725 and its mutants were cultured at 30°C in an orbital shaker at 200 rpm using NB as rich medium supplemented with 2 mM E2, 2 mM E1, or 1.89 mM E3. Estrogens were added from a stock solution prepared in CDX as described above.

### DNA Manipulation

DNA manipulation protocols were performed as described elsewhere (Sambrook and Russell, 2001). *N. tardaugens* genomic DNA was extracted as described before (Ibero et al., 2019b). Plasmid DNA was purified using High Pure Plasmid Isolation Kit (Roche). DNA fragments were purified with QIAquick PCR Purification Kit (Qiagen) or QIAquick Gel Extraction Kit (Qiagen). *E. coli* cells were transformed using the RbCl method (Sambrook and Russell, 2001) or by electroporation using a Gene Pulser (Bio-Rad) (Wirth et al., 1989). DNA amplification was performed in a Mastercycler Gradient (Eppendorf) using the oligonucleotides listed in Table S2, which were purchased from Sigma (Germany). Phusion High-Fidelity DNA Polymerase (New England Biolabs) was used for cloning amplifications and Taq DNA polymerase (Biotools) for screening. All PCR products were checked by agarose gel electrophoresis and those aimed for cloning were confirmed by DNA sequencing by Secugen S.L. (Spain). Digestion of DNA fragments was done using restriction enzymes (New England Biolabs), and ligation was performed with Instant Sticky-end Ligase Master Mix (New England Biolabs).

### RNA Extraction

Total RNA of *N. tardaugens* cells was extracted from cultures grown on minimal medium with 20 mM CDX and E2 or PYR as carbon sources as described previously (Ibero et al., 2019a). Briefly, cells where harvested in mid exponential phase (OD_600_ 0.6) and stored at −80°C. Pellets where thawed and cells were lysed in 400 μl TE buffer (10 mM Tris–HCl, 1 mM EDTA, pH 7.5) containing lysozyme (50 mg/ml) following three freezing–thawing cycles. High Pure Isolation Kit (Roche), followed by DNA-free DNA Removal Kit (Invitrogen) treatment, was used to obtained pure RNA. Purity and concentration were measured in a ND1000 spectrophotometer (Nanodrop Technologies).

### Transcriptomic Analysis (RNA-Seq)

RNA-seq was done in Macrogen Korea. Total RNA integrity was checked using an Agilent Technologies 2100 Bioanalyzer. Ribosomal RNA was removed from the total RNA with Ribo-Zero rRNA Removal Kit to later construct a 100-bp paired-end library using TruSeq RNA Sample Prep Kit v2 that was quality-checked in an Agilent Technologies 2100 Bioanalyzer using a DNA 1000 chip. Library sequencing was performed in a HiSeq 3000 4000 (Illumina) using TruSeq 3000 4000 SBS Kit v3 as reagent. Bioinformatics analyses were performed by the Bioinformatics and Biostatistics Service of the Center for Biological Research Margarita Salas (CIBMS-CSIC). Raw read data quality was checked using FastQC and trimmed with Trimmomatic. Trimmed reads were mapped against the genome sequence of *N. tardaugens* (accession number CP034179) using Bowtie2, and expression quantification was done using HTSeq-count. An average of 69 million raw sequencing reads (approximately 6.9 billion base pairs; average 1,600× genome coverage per sample) were generated from samples from two independent experiments in the presence of PYR or E2, each with three biological replicates. After trimming the raw sequence reads, an average 25.3 million high-quality clean reads were mapped to the *N. tardaugens* reference genome and between 74.4 and 59.3% were uniquely mapped. Differential expression analysis was done using Deseq2. The dissimilarity matrix shown in the heatmap was obtained with the Euclidean distance, and the cluster analysis was performed with Ward's minimum variance method. Bioinformatics analysis software was used with default settings. Raw read data obtained from the three replicates of the transcriptome of the strain grown on PYR and E2 have been deposited in the Sequence Read Archive (SRA) database of the National Center for Biotechnology Information (NCBI) under accession numbers SRR9027780, SRR9027781, and SRR9027779 (Bioproject PRJNA541800) and in the European Nucleotide Archive (ENA) database of the European Bioinformatics Institute (EMBL-EBI) under accession number ERP122552 (BioprojectPRJEB39081), respectively.

### Construction of *N. tardaugens* Knockout Strains

The knockout strains were constructed by double homologous recombination using the suicide vector pK18mobsacB (Schafer et al., 1994) as described before (Ibero et al., 2019a). *N. tardaugens* genomic DNA was used as template to amplify two fragments of ≈700 bp containing the upstream and downstream regions of the gene to delete UP and DOWN fragments (Table 1 and Table S2), respectively. The fragments were digested with the appropriate restriction enzymes and cloned in the unique sites of the plasmid. The ligation product was transformed into *E. coli* DH10B-competent cells, and once recombinant candidates were PCR-checked, the cloned region was confirmed by sequencing. The plasmids were transformed by triparental conjugation (Herrero et al., 1990) into *N. tardaugens* Rf^R^ as recipient strain using *E. coli* HB101 (pRK600) (Sharma and Signer, 1990) as helper and *E. coli* DH10B, harboring the corresponding vector, as donor. To prepare the recipient strain, 10 ml of late exponential phase cultures were centrifuged at 13,000 rpm for 1 min, and the pellet was washed with one volume of sterile 0.85% NaCl solution. The cells were centrifuged again and the pellet was resuspended to a final volume of 100 μl of 0.85% NaCl solution. One milliliter of overnight cultures of donor and helper strains were centrifuged at 13,000 rpm for 1 min, and the pellet was washed in 500 μl of sterile 0.85% NaCl solution. Fifty microliters of each strain was mixed and pipetted to a 0.22-μl filter disc placed on the NB plate. The plate was incubated overnight at 30°C. The next day, the filter mating disks were collected in a 1.5-ml tube with 1 ml of a sterile 0.85% NaCl solution and vortexed thoroughly to detach the cells from the filter. Afterwards, 100 μl and the rest of the cells were plated on NB plates containing selective antibiotic kanamycin (10 μg/ml) and rifampicin (50 μg/ml) and screened by PCR using appropriate primers (Table S2). Selected candidates were grown up to stationary phase (≈48 h) in NB medium and then plated in NB supplemented with 5% sucrose. The clones that are resistant to sucrose and sensitive to kanamycin were checked by PCR using external primers, and the amplicon was sequenced to confirm the second crossover event.

### Complementation of *N. tardaugens* Knockout Strains

The mutant strains were transformed with a pSEVA23 plasmid harboring the corresponding deleted gene under the expression control of *P*_*lexA*_ constitutive promoter (Fernández et al., 2014; Table 1) using triparental conjugation, following the same protocol as used to construct *N. tardaugens* knockout strains. The *P*_*b*_ native promoter from *N. tardaugens* was also used for gene expression in pSEVA23 plasmid and corresponds to a region of 300 pb upstream of *EGO55_13570* gene which includes the intergenic region of the *edc* cluster, where the putative promoters of the cluster are located.

### Organic Phase Extraction and Thin Layer Chromatographic Analysis

The presence of steroidal compounds in culture media and enzymatic assay mixtures was determined after organic solvent extraction by thin layer chromatographic (TLC) analysis as described before (Ibero et al., 2019a). Two volumes of chloroform were added, and the mixture was vortexed for 30 s and centrifuged for 1 min at 13,000 rpm in an Eppendorf microcentrifuge. The organic phase was extracted and dried. The dried sample was dissolved in 100 μl of acetonitrile and analyzed by thin layer chromatography (TLC). For TLC analysis, 10 μl of the standards and the samples dissolved in acetonitrile were spotted in silica gel plates (TLC Silicagel 60 F_2_54, Merck Millipore), and *n*-hexane:ethyl acetate (10:4 v/v) was used as a developing system. Steroid products were visualized by UV and revealed by spraying 20% (v/v) sulfuric acid and heating at 120°C.

### HPLC-MS and GC-MS Analysis

Detection and identification of different steroids extracted from cultures (Table 2) and from the hydroxylation reaction carried out with EdcA was carried using high-efficiency liquid chromatography coupled to a photodiode array detector and mass spectroscopy (HPLC-DAD-MS) using liquid chromatography equipment (Surveyor Plus LC) equipped with automatic injector coupled to a diode array detector (DAD) and with an ion trap (LXQ) equipped with a source of electrospray ionization (ESI), all supplied by Thermo Electron (San Jose, CA, United States). The data was processed with the Xcalibur program (Thermo Fisher Scientific, San Jose, CA, United States). Chromatographic separation was performed using a Mediterranea Sea C18 reverse phase column (150 × 4.6 mm internal diameter, 5 μm particle size) (Teknokroma, Barcelona, Spain). Samples were eluted with a 1 ml min^−1^ gradient flow (solvent A, H_2_O + 0.1% formic acid; solvent B, CH_3_CN + 0.1% formic acid) starting with 30% B for 5 min and increasing solvent up to 55% B for 20 min. The solvent B % was then increased to 100% in 5 min. The elution was maintained at 100% B for 5 min and then reduced again to 30% B in 5 min, followed by equilibration of the column for 5 min prior to injection of the next sample.

**Table 2:** LC-MS analysis of metabolites involved in estrogen catabolism in *N. tardaugens*.

**Compound name**	**LC-MS RT (min)**	**Molecular formula**	**Dominant ion peaks**	**Identified product ions**
E2	18.39	C_18_H_24_O_2_	255.15	[M–H_2_O+H]^+^
E1	22.14	C_18_H_22_O_2_	271.19	[M+H]^+^
4-OHE1	17.06	C_18_H_22_O_3_	287.27	[M+H]^+^
Pyridinestrone acid	3.28	C_18_H_21_O_3_N	300.21	[M+H]^+^
4-Norestrogen-5(10)-en-3-oic acid (M5 deconjugated)	12.76	C_17_H_22_O_4_	273.32	[M–H_2_O+H]^+^
E3	5.55	C_18_H_24_O_3_	271.19	[M–H_2_O+H]^+^
16-keto-E2/16α-OH-E1	9.65	C_18_H_22_O_3_	287.31	[M+H]^+^
4-OH-16-keto-E2/4-OH-16α-OH-E1	5.79	C_18_H_22_O4	303.28	[M+H]^+^

The enzymatic reactions were monitored by HPLC using an Agilent Series 1200 HPLC system and the same reverse phase C18 column. The samples were eluted isocratically at a flow rate of 1 ml min^−1^ (solvent A, H_2_O + 0.1% FA; solvent B, CH_3_CN + 0.1% FA) with a gradient starting at 60% B up to 2 min, and the solvents ramped up to 100% B over 9 min. The elution was maintained at 100% B for 2 min and then ramped back to 60% B within 2 min, followed by equilibration at the same composition for 2 min before the next run. The elution was monitored at 240 nm. When required, the enzymatic assays were monitored by GC-MS. The chloroform extracted fraction was concentrated by evaporation, and the trimethylsilyl ether derivatives were formed by reaction with 50 μl of BSTFA and 50 μl of pyridine and heating at 60°C for 45 min. Calibration standards were derivatized in the same way. The GC/MS analysis was carried out using an Agilent 7890A gas chromatograph coupled to an Agilent 5975C mass detector (Agilent Technologies, Palo Alto, CA, United States). Mass spectra were recorded in electron impact (EI) mode at 70 eV within the *m*/*z* range 50–550. The chromatograph was equipped with a 30-m × 0.25-mm i.d. capillary column (0.25 m film thickness) HP-5MS (5% diphenyl 95% dimethylpolysiloxane from Agilent Technologies). Working conditions in the sample were as follows: split ratio 20:1, injector temperature 320°C, and column temperature 240°C for 3 min, then heated to 320°C at 5°C min^−1^. EI mass spectra and retention data were used to assess the identity of compounds by comparing them with those of standards in the NIST Mass Spectral Database and commercial standards.

### Cloning and Expression of CYP450

The DNA fragment containing the *EGO55_13525* gene was amplified by PCR with primers specified in Table S2, digested with the corresponding restriction enzymes and then ligated into the pET29a vector yielding pET29-edcA. Electrocompetent cells of *E. coli* BL21 (DE3) were transformed with pETedcA. The resulting strain *E. coli* BL21 (DE3) (pETedcA) was cultured in 50 ml LB containing kanamycin up to an OD_600_ of 0.5–0.8, and gene expression was induced with 0.2 mM IPTG and aminolevulinic acid. After 3 h of induction, the cells were harvested, washed with 0.85% saline solution, and resuspended in 20 mM Tris–HCl (pH 8.0). Cells were lysed by sonication using a Branson sonicator applying three cycles of 30-s bursts at maximum power alternated with 30 s cooling in ice. Soluble fraction was separated by centrifugation in a Sorvall Linx 6000 (SS-34 rotor, 15 min at 4°C and 14,000 rpm), and protein concentration was calculated by the Bradford method (Bradford, 1976). The overproduction of EdcA in the soluble fraction of the crude extract was checked by SDS-polyacrylamide gel electrophoresis.

### Enzymatic Assay of E1-Hydroxylase Activity

The enzymatic activity of CYP450 was examined *in vitro* using heterologous electron donor partners: spinach ferredoxin and ferredoxin reductase and a NADPH regenerating system, as described previously (García-Fernández et al., 2013). Reactions were carried out in glass tubes at ambient temperature in a volume of 200 μl. Estrogen stock solutions (10 mM) were prepared in methanol. Crude extract (70 μg of protein) was pre-incubated 5 min with 100 μM of substrate in 50 mM sodium phosphate buffer (pH 7.5) containing 0.45% (w/v) MβCD and 10 mM MgCl_2_. Reactions were initiated by adding 0.3 mM NADPH, 1 μM spinach ferredoxin, 0.2 U ml^−1^ spinach ferredoxin-NADP^+^ reductase, 0.1 mg ml^−1^ bovine liver catalase, and an NADPH-regenerating system consisting of 0.4 U ml^−1^ glucose 6-phosphate dehydrogenase and 5 mM glucose 6-phosphate. Aliquots of 50 μl were taken at 0, 5, and 30 min and quenched with 150 μl of acetonitrile containing 0.1% formic acid (FA). The reaction mixtures were centrifuged at 10,000×*g* for 4 min.

### *In silico* Analysis

Protein sequence comparisons where made using the Standard Protein Basic Local Alignment Search Tool (BLASTp) (Altschul et al., 1990). The HMG-CoA synthetases sequences were collected from the public databases. The sequences were aligned by Clustal Omega built on Geneious Prime (available from http://www.geneious.com). The neighbor-joining analysis was performed using MEGA7 (Kumar et al., 2018).

## Conclusion

The present study constitutes a step forward in understanding estrogen catabolism in bacteria. The transcriptome of *N. tardaugens* cells grown on E2 revealed a conserved estrogen degradation gene cluster, named *edc*. Genetic tools were applied in this estrogen-degrading bacterium to delete and complement some of the genes of the *edc* cluster to determine their precise role. These tools will allow elucidating the complete biochemical reactions of estrogen degradation in the near future. One of the main findings has been that, as it occurs in mammals, a CYP450 encoded by the *edcA* gene performs the 4-hydroxylation of estrone in this strain. Subsequent degradation steps, like A-ring cleavage of 4-OHE1 by a 4,5-dioxygenase (EdcB), and decarboxylation of the resulting alkylic chain by an indolepyruvate ferredoxin oxidoreductase (EdcC), have been confirmed *in vivo*. Moreover, we have linked steroid uptake to a TonB-dependent receptor protein (EdcT) conserved among estrogen degraders that appears to be necessary to achieve efficient estrogen mineralization in *N. tardaugens*.

## Data Availability Statement

The datasets generated for this study can be found in Sequence Read Archive (SRA) 26 database of the National Center for Biotechnology Information (NCBI) under accession numbers SRR9027780, SRR9027781, SRR9027779 (Bioproject PRJNA541800), and in the European Nucleotide Archive (ENA) database of the European Bioinformatics Institute (EMBL-EBI) under accession number ERP122552 (BioprojectPRJEB39081).

## Author Contributions

JI carried out the experiments and helped in drafting the manuscript. JG and BG conceived and coordinated the study and drafted the manuscript. JG, JI, BG, and VR-B participated in analyzing the data. All authors read, reviewed, and approved the final manuscript.

## Conflict of Interest

The authors declare that the research was conducted in the absence of any commercial or financial relationships that could be construed as a potential conflict of interest.
