# Supplementary material for: Unraveling the 17β-Estradiol Degradation Pathway in Novosphingobium tardaugens NBRC 16725
Source: Front Microbiol. 2020 Dec 7;11:588300. doi: 10.3389/fmicb.2020.588300 (PMC7793797; doi:10.3389/fmicb.2020.588300)
Supplement: Supplementary file 1 [file Data_Sheet_1.docx]

**Table S1.** Gene expression analysis (RNA-seq) of *N. tardaugens* grown on E2 condition compared to PYR. Genes located in the *edc* cluster (purple and pink), the SD cluster (green) and those involved in methylmalonyl-CoA pathway (yellow) and cofactor B12 biosynthesis pathway (orange) are highlighted.

**Table S2. Primers used in this study.** Restriction enzymes used for cloning are shown in brackets.

| **Primers** | **Sequence (5'-3')** | **Use** |
| --- | --- | --- |
| 5NdeIpCWCYPX450f | CTAGCATATGGCTGCCGCTTACGACCCCTT | Cloning *EGO55_13525* into pET29a(+) (*Nde*I/*Sal*I) |
| 3SalIpCWCYPX450r | CTAGGTCGACTCAGTGGTGGTGGTGGTGGTGCTTCCAGCGCGGCCTGATC |  |
| 5'UPXbaIIntGenF | CACATTCTAGATGGCGATTTTCATGGCGG | Amplification of fragment UP-Prom for ΔProm construction (*Xba*I/*Sal*I) |
| 3'UPSalIIntGenR | ACATGTCGACCGGGATGGACCTCAGCA |  |
| 5'DOWNSalIIntGenF | ACGCGTCGACGGGAGAGGTGCATGGAA | Amplification of fragment DOWN-Prom for ΔProm construction (*Sal*I/*Hin*dIII) |
| 3'DOWNHindIIIIntGenR | ACATAAGCTTAAGCCTGCCCCACCATG |  |
| 5'UPXbaIOpUpF | CACATTCTAGAAGTTTTCGAGCTTGCGGA | Amplification of fragment UP-OpA for ΔOpA construction (*Xba*I/*Sal*I) |
| 3'UPSalIOpUpR | ACATGTCGACTTAGGCAGGGCATCGGG |  |
| 5'DOWNSalIOpUpF | ACGCGTCGACTCCCGCTCTTCTCCCA | Amplification of fragment DOWN-OpA for ΔOpA construction (*Sal*I/*Hin*dIII) |
| 3'DOWNHindIIIOpUpR | ACATAAGCTTCCGAGCAGGCGCGTATA |  |
| 5'UPXbaIOpDoF | CACATTCTAGAACCATGCGTTCTGCCCC | Amplification of fragment UP-OpB for ΔOpB construction (*Xba*I/*Sal*I) |
| 3'UPSalIOpDoR | ACATGTCGACCCATGCACCTCTCCCAA |  |
| 5'DOWNSalOpDoF | CACATGTCGACAGTTGCTTTCCTTCCGGC | Amplification of fragment DOWN-ΔOpB for ΔOpB construction (*Sal*I/*Sph*I) |
| 3'DOWNSphIOpDoR | ACATGCATGCGCGGCAAGGCATCGAGA |  |
| 5_GIBCYPX450EcoRIUPf | ATGACATGATTACGAATTCCCCGATGCCCTGCCTAAC | Amplification of fragment UP-edcA for ΔedcA construction (*Eco*RI/*Bam*HI) |
| 3_GIBCYPX450BamHIUPr | CTGACCTTGGGATCCATCAGGCCGCGCTGGAAGTA |  |
| 5_GIBCYPX450BamHIDOWNf | CGGCCTGATGGATCCCAAGGTCAGTCTCCGGGAAG | Amplification of fragment DOWN-edcA for ΔedcA construction (*Bam*HI/*Hin*dIII) |
| 3_GIBCYPX450HindIIIDOWNr | ACGGCCAGTGCCAAGCTTGCTTCGTTCCGCGTGGC |  |
| 5EcoRIDioxUPf | TATACGAATTCTCGACCATATCAGCATCGGC | Amplification of fragment UP-edcB for ΔedcB construction (*Eco*RI/*Bam*HI) |
| 3BamHIDioxUPr | CACACGGATCCGCCCAGACCGAGAATTTCCA |  |
| 5BamHIDioxDOWNf | CACACGGATCCAGCATCGAGGAAACGGGAC | Amplification of fragment DOWN-edcB for ΔedcB construction (*Bam*HI/*Hin*dIII) |
| 3HindIIIDioxDOWNr | CACACAAGCTTTACTTCGCCTTGCCGATCAA |  |
| 5_GIBIndoleEcoRIUPf | TATGACATGATTACGAATTCTCGACGGGGTACACCATGT | Amplification of fragment UP-edcC for ΔedcC construction (*Eco*RI/*Bam*HI) |
| 3_GIBIndoleBamHIUPr | GCCACCTGGGATCCCGTCATGCGGCGGCC |  |
| 5_GIBIndoleBamHIDOWNf | GCATGACGGGATCCCAGGTGGCCGATGCCC | Amplification of fragment DOWN-edcC for ΔedcC construction (*Bam*HI/*Hin*dIII) |
| 3_GIBIndoleHindIIIDOWNr | GCCAGTGCCAAGCTTACACTTCGTTGGTCCAGTGA |  |
| 5EcoRITonBUPf | ATATCGAATTCTTCTGGTCGAACTGGGTGAAC | Amplification of fragment UP-edcT for ΔedcT construction (*Eco*RI/*Bam*HI) |
| 3BamHITonBUPr | CACACGGATCCCGCATAGATACCTCCCTGGG |  |
| 5BamHITonBDOWNf | CACACGGATCCCGAGGTCAAAGTCGCCTTCT | Amplification of fragment DOWN-edcT for ΔedcT construction (*Bam*HI/*Hin*dIII) |
| 3HindIIITonBDOWNr | CACACAAGCTTGGGATGCTTCCACACATCGA |  |
| F24 | CGCCAGGGTTTTCCCAGTCACGAC | Internal primer: used to check insert cloning in pK18*mob*sacB and to check insertion of pK18*mob*sacB |
| R24 | AGCGGATAACAATTTCACACAGGA |  |
| ExtIntGenF | GCCAGAACCGTGCTTTCG | External primer: together with F24 used to check insertion of pK18Prom. Together with ExtIntGenR used to check the deletion of the intergenic región between *EGO55_13565* and *EGO55_13570* |
| ExtIntGenR | GGATGCACAGCAAGGCG | External primer: together with R24 used to check insertion of pK18Prom. Together with ExtIntGenF used to check the deletion of the intergenic región between *EGO55_13565* and *EGO55_13570* |
| ExtOpUpF | CGCGCGGGTGACTTGTA | External primer: together with F24 used to check insertion of pK18OpA. Together with ExtOpUpR used to check deletion of the region *EGO55_13565*-*EGO55_13520* |
| ExtOpUpR | GTCTGATCCGGTCCGACA | External primer: together with R24 used to check insertion of pK18OpA. Together with ExtOpUpF used to check deletion of the region *EGO55_13565*-*EGO55_13520* |
| ExtOpDoF | GGCCATGGAAAGATCGGC | External primer: together with F24 used to check insertion of pK18OpB. Together with ExtOpDoR used to check deletion of the region *EGO55_13570*-*EGO55_13600* |
| ExtOpDoR | CTGCGTCATCCAGGTCCA | External primer: together with R24 used to check insertion of pK18OpB. Together with ExtOpDoF used to check deletion of the region *EGO55_13570*-*EGO55_13600* |
| CYPX450EXTf | CATGACAGGACCACGTCACA | External primer: together with F24 used to check insertion of pK18edcA. Together with CYPX450EXTr used to check *EGO55_13525* deletion |
| CYPX450EXTr | GTCGATGTGGCGCAGATCTA | External primer: together with R24 used to check insertion of pK18edcA. Together with CYPX450EXTf used to check *EGO55_13525* deletion |
| 5ExtDioxf | TGACGTTGGCCATGGAAAGA | External primer: together with F24 used to check insertion of pK18edcB. Together with 3ExtDioxr used to check *EGO55_13570* deletion |
| 3ExtDioxr | CCGGCATGCCAAAATGGATC | External primer: together with R24 used to check insertion of pK18edcB. Together with 3ExtDioxf used to check *EGO55_13570* deletion |
| IndoleEXTf | GGCACATCAATCTGTTCGCC | External primer: together with F24 used to check insertion of pK18edcC. Together with IndoleEXTr used to check *EGO55_13580* deletion |
| IndoleEXTr | GCAAACATAGGTCCAGCCCT | External primer: together with R24 used to check insertion of pK18edcC. Together with IndoleEXTf used to check *EGO55_13580* deletion |
| 5ExtTonBf | GCTCGCCCTGCATCATCTTA | External primer: together with F24 used to check insertion of pK18edcT. Together with 3ExtTonBr used to check *EGO55_13555* deletion |
| 3ExtTonBr | AATATCCATCGCGCGGTACC | External primer: together with R24 used to check insertion of pK18edcT. Together with 5ExtTonBf used to check *EGO55_13555* deletion |
| T0pSEVA237F | CCGAGCGTTCTGAACAAATCC | Internal primer: Together with R24, used to check insert cloning in pSEVA237PlexA. Hybridizes in T0 terminator. |
| 3SEQIntRegGFPr | ACATTTAATTAAACTCCAGTGAAAAGTTCTTCTCCT | Internal primer: Together with R24, used to check insert cloning in pSEVA237PlexA. Hybridizes in *gfp* gene |
| 5XbaICOMPCYPX450F | CACATTCTAGAAGGAGGAAAAACATTTGGCTGCCGCTTACG | Cloning *EGO55_13525* into pSEVA237PlexA (*Xba*I/*Spe*I) |
| 3SpeICOMPCYPX450R | CACACACTAGTCTACTTCCAGCGCGGC |  |
| 5XbaICOMPDioxF | CACATTCTAGAAGGAGGAAAAACATATGGAAATTCTCGGTCTGGGC | Cloning *EGO55_13570* into pSEVA237PlexA (*Xba*I/*Spe*I) |
| 3SpeICOMPDioxR | CACACTCAACTAGTTCAAGCCATTACGCCCTCCG |  |
| 5BamHICOMPIndole2F | ATACGGATCCAGGAGGAAACATATGACGTTTTCTCTCGACGA | Cloning *EGO55_13580* into pSEVA237PlexA (*Bam*HI/*Spe*I) |
| 3SpeICOMPIndole2R | CACATACTAGTGCGGGATTCTTCGGCAGA |  |
| 5BamHICOMPTonB2F | ATACGGATCCAGGAGGAAACATATGCGTTTTCGTGAGC | Cloning *EGO55_13600* into pSEVA237PlexA (*Bam*HI/*Spe*I) and into Zero Blunt® TOPO® vector |
| 3SpeICOMPTonB2R | CACATACTAGTCGGGCGGTGCTTCTCAA |  |
| 5EcoRIStopIntReg1f | ATATCGAATTCCTAATCGTCGTAGATGGCGGAAAC | Cloning Pb promoter region into pSEVA237PlexA (*Eco*RI/*Sal*I) |
| 3SalIIntReg1r | ACATGTCGACAAAAAACGCACTTTCGATACTATTGGT |  |
| 5EcoRICSSCYPX450F | ATACTGAATTCTGACCTAAGGAGGTAACAATTGGCTGCCGCTTACG | Cloning *EGO55_13525* into pSEVA237PbPlexA (*Eco*RI/*Bam*HI) |
| 3BamHICSSCYPX450R | CACATGGATCCCTTAAGCTACTTCCAGCGCGGC |  |
| 5AflIICSSDioxF | ATACTCTTAAGGCTAGCCTAAGGAGGTAACATATGGAAATTCTCGGTCTGGGC | Cloning *EGO55_13570* into pSEVA237Pb-edcA (*Afl*II/*Xba*I) |
| 3XbaIStopCSSDioxR | CACATTCTAGAGGGCCCTCAAGCCATTACGCCCTCCG |  |
| 5XbaICSSIndoleF | CACATTCTAGATGACCTAAGGAGGTAACATATGACGTTTTCTCTCGACGA | Cloning *EGO55_13580* into pSEVA237Pb-edcA-edcB (*Xba*I/*Hin*dIII) |
| 3HindIIICSSIndoleR | ACATAAGCTTCTCGAGTCAGCGGGATTCTTCGGCAGA |  |

**Figure S1.** Gene expression level profile observed in E2 and PYR growth conditions. Heatmap diagram of cluster analysis showing the log2 mean normalize expression in each experimental growth condition for those genes with FC > 2 and FC < -2


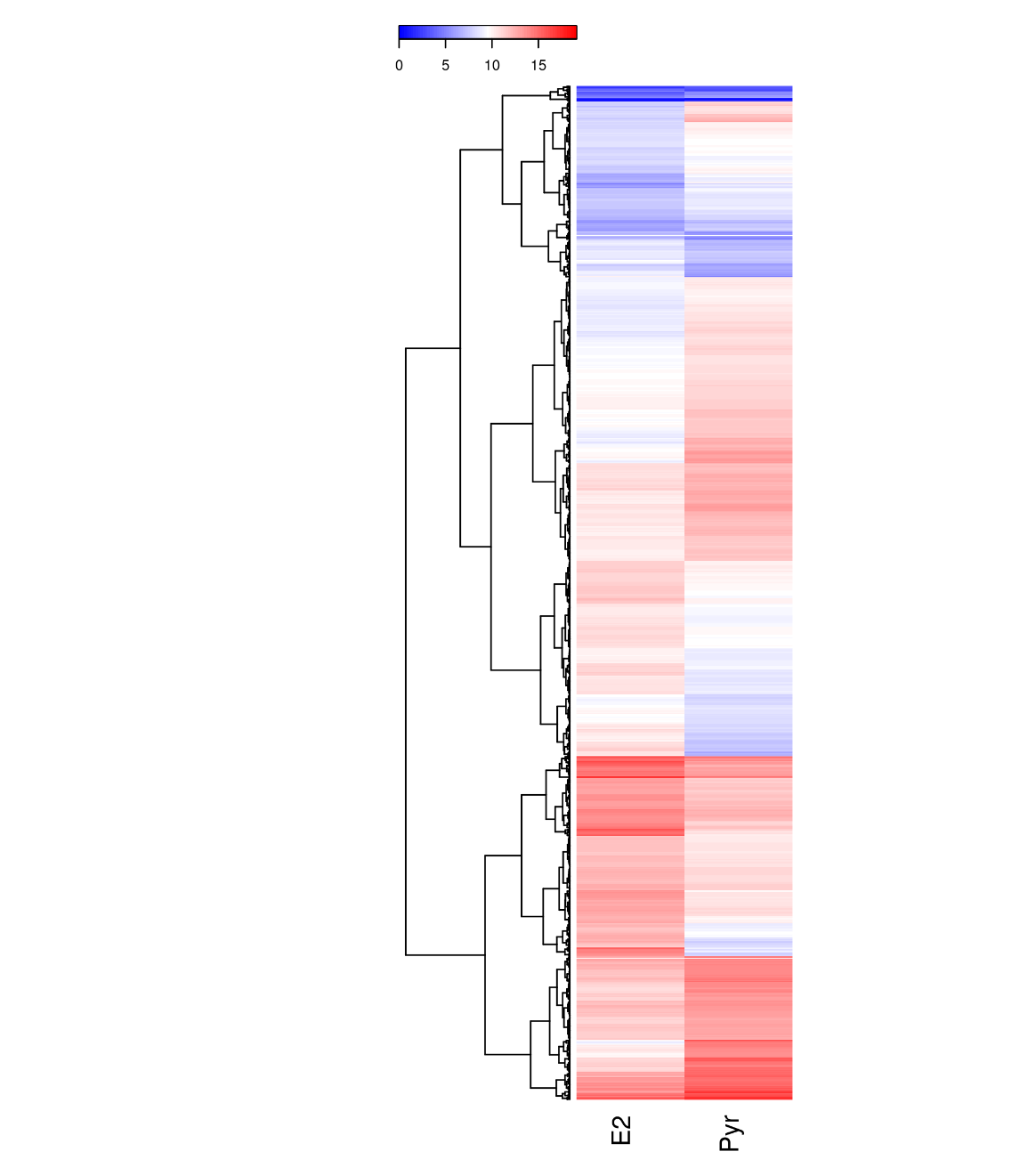


**Figure S2.** TLC analysis of the organic phase extracted from cultures of *N. tardaugens* NBRC 16725 (blue), ΔProm (yellow), ΔOpA (red) and ΔOpB (purple) strains in NB rich medium supplemented with 2 mM E2 (red arrow indicates 4-OHE1). E2 and E1 standards, in a 1 mM concentration, are also shown.

**ΔProm**

**wt**

**ΔOpA**

**ΔOpB**

**ΔProm**

**wt**

**ΔOpA**

**ΔOpB**

**ΔProm**

**wt**

**ΔOpA**

**ΔOpB**

**0 h**

**16 h**

**24 h**


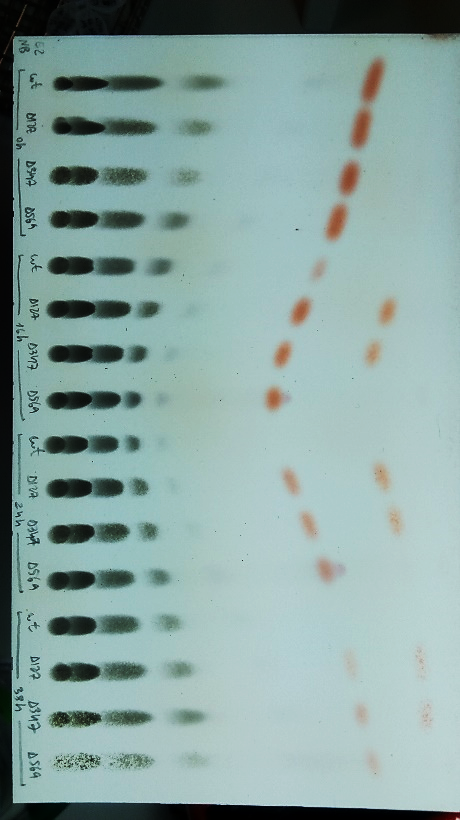


**E1**

**E2**

**Figure S3.** 12.5% SDS-polyacrylamide gel electrophoresis of the overproduction of EdcA proteins in the soluble fraction of the crude extract of *E. coli* BL21(DE3) strains. 25 µg of total protein of each sample where loaded. **1.** Molecular weight size marker. **2.** Protein extract from *E. coli* BL21 (De3) (pET29-ecdA). Black arrow indicates EdcA (44.29 kDa).

**Figure S4.** GC-MS analysis of the CYP450 enzymatic assays using BL21 (DE3) (pETedcA) cells crude extract with E1 **(a)** and E2 **(b)** as substrates. **(c)** Chemical structure and fragmentation pattern of 4-OHE2.

3.00

4.00

5.00

6.00

7.00

8.00

9.00

10.00

11.00

12.00

5000

10000

15000

20000

25000

30000

35000

40000

45000

50000

55000

60000

65000

70000

75000

80000

85000

90000

95000

Estrone

4-OHE1

3.00

4.00

5.00

6.00

7.00

8.00

9.00

10.00

11.00

12.00

5000

10000

15000

20000

25000

30000

35000

40000

45000

50000

55000

60000

65000

70000

75000

80000

85000

90000

95000

Time-->

Abundance

Estrone

**(a)**

3.00

4.00

5.00

6.00

7.00

8.00

9.00

10.00

11.00

12.00

20000

40000

60000

80000

100000

120000

140000

160000

180000

200000

Abundance

Estradiol

3.00

4.00

5.00

6.00

7.00

8.00

9.00

10.00

11.00

12.00

20000

40000

60000

80000

100000

120000

140000

160000

180000

200000

8.371

Estradiol

4-OHE2

Time-->

**(b)**


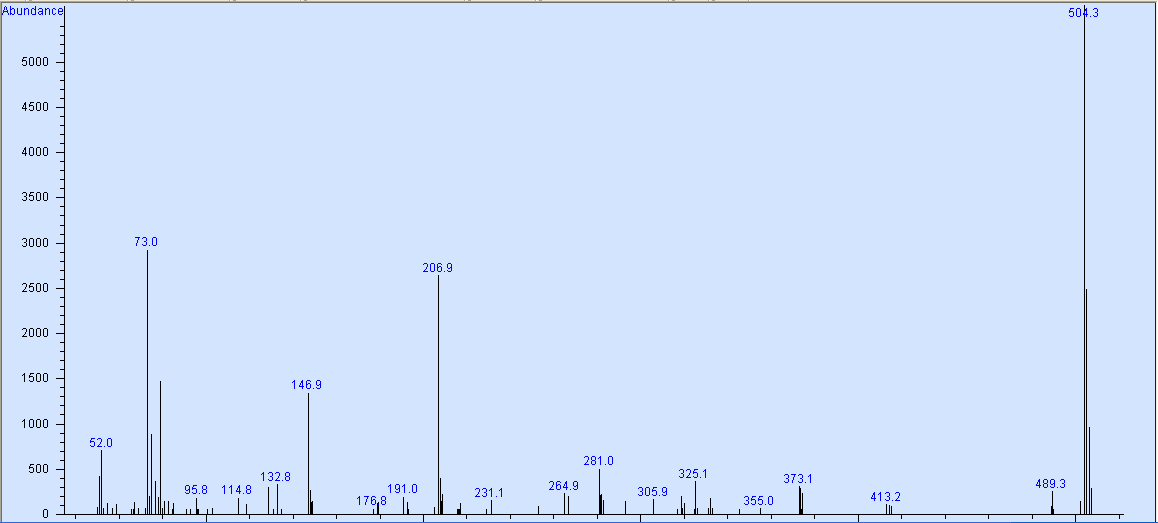


**(c)**

**Figure S5.** Diagrams of plasmids constructed for *in vivo* characterization of EdcA, EdcB y EdcC. **(a)** pSEVA237-Pb-edcA, **(b)** pSEVA237-Pb-edcAB and **(c)** pSEVA237-Pb-edcABC. Pb, intergenic region of *edc* cluster; T0 y T1, transcription terminators; Km^R^, kanamycin resistance gene; *OriT*, origin of transference and *OriV* (pBBR1), origin of replication. Restriction enzymes used for cloning are also indicated.

**Figure S6**. Phylogenetic tree of HMG-CoA synthases. Accession numbers are as follows: *Altererythrobacter estronivorus* (WP_067531102.1), *Arabidopsis thaliana* (AA58763.1), *Aspergillus fumigatus* (KEY82524.1), *Candida albicans* (AOW31581.1), Delta proteobacterium (TDJ11724.1), *Desulfurococcus amylolyticus* (AFL66276.1), *Enterococcus faecalis* (EPI22672.1), *Enterococcus faecium* (EJX57065.1), HUMAN, human cytoplasmic enzyme (Q01581), *Methanoplanus limicola* (EHQ34993.1), MIT, human mitochondrial enzyme (NP005509), *Myxococcales* bacterium (HIF97404.1), *Novosphingobium tardaugens* (WP_021690444.1), *Pinus sylvestris* (CAA65250.1), *Polyangiaceae* bacterium (NRA32649.1), *Pseudomaricurvus alkylphenolicus* (WP_166987910.1), *Rattus novergicus* (MITP22791.2), *Sacharomyces cerevisiae* (P54839), *Shingobium estronivorans* (WP_150291465.1), *Sphingomonas* sp. strain KC8 (WP_010123502.1), *Spirochaeta* sp. (MAI26667.1), *Staphylococcus aureus* (OBX95572.1), *Staphylococcus haemolyticus* (RFU01746.1), *Streptococcus epidermidis* (KAB1899410.1), *Streptococcus pneumoniae* (ABJ53679.1), *Streptococcus pyogenes* (KAB1890604.1), *Thermosphaera aggregans* (ADG90969.1), *Trichoderma reesei* (XP006962798.1) and *Yarrowia lipolytica* (Q6BZW0).
